# The Fate of Per- and Polyfluoroalkyl Substances (PFAS) in Municipal Solid Waste Incineration: Atmospheric Emissions from a Waste-to-Energy Plant

**DOI:** 10.1021/acs.est.6c05879

**Published:** 2026-10-20

**Authors:** Jelle Hofman, Jan Peters, Griet Jacobs, Aline Reis De Carvalho, Gill Van den Bergh, Bart Baeyens, Wim Aerts, Jo Van Laer, Nic Moonen, Wouter Lefebvre, Patrick Berghmans, Stefan Voorspoels, Gert Otten

**Affiliations:** † Air Quality & Industrial Emissions (AQIE) Team, 54520 Flemish Institute for Technological Research (VITO), Boeretang 200, Mol 2400, Belgium; ‡ Specialized Organic and Inorganic Analysis Lab (GOAL), 54520 Flemish Institute for Technological Research (VITO), Boeretang 200, Mol 2400, Belgium; § Geology & Air Quality Modelling (GAM) Team, 54520 Flemish Institute for Technological Research (VITO), Boeretang 200, Mol 2400, Belgium

**Keywords:** PFAS, waste to energy, WtE, emissions, flue gas, waste

## Abstract

The atmospheric fate of per- and polyfluoroalkyl substances (PFAS) represents a very recent field of research. While ISO and CEN standards are yet under development, PFAS have been identified during the past years in atmospheric emissions, ambient air, and deposition. As high-quality field data are currently scarce, the objectives of this study were to (i) characterize atmospheric PFAS emissions of a state-of-the-art waste-to-energy (WtE) plant under representative operational conditions and (ii) evaluate the emission impact in ambient air in the vicinity of the plant. Results suggest a contribution from the WtE plant at the nearest ambient monitoring location, 450 m downwind of the plant, with a measurable contribution and distinct PFAS fingerprint closely matching the emission profiles independently characterized at two grate incinerator lines. Quantified PFAS emissions do not exceed the temporary assessment framework for ambient air in Flanders. Furthermore, optimization of the activated carbon (AC) dosage achieved a 95% reduction in emissions at line 1, with subsequent emission levels approaching those of field blanks. The waste treatment sector represents a critical frontier of environmental management. Building experimental evidence of potential PFAS releases will be key to ensure comprehensive strategies for responsible PFAS management in the years ahead.

## Graphical abstract

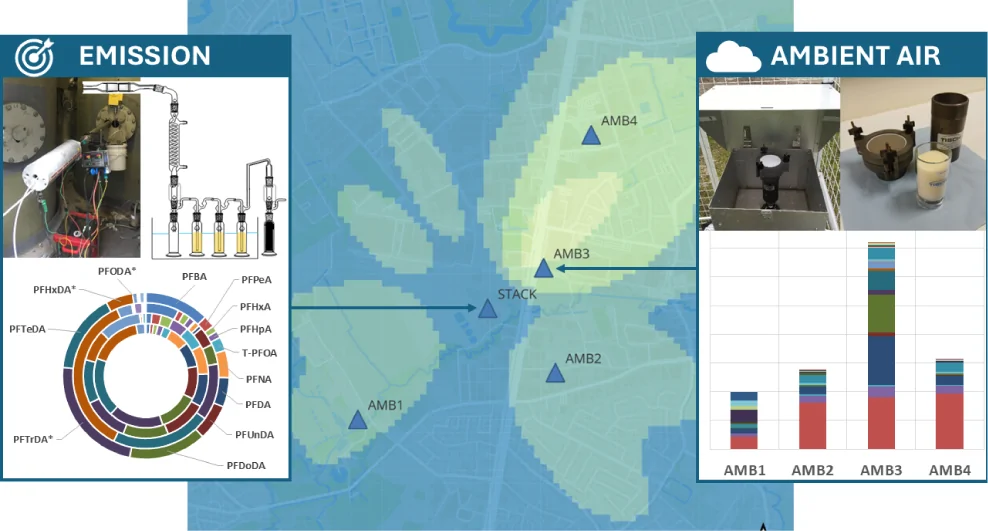

## 1 Introduction

PFAS are a large class of >10,000 synthetic chemicals whose persistence and mobility in the environment have raised global concern.^1,2^ While extensive research has focused on PFAS exposure through soil, food, and water,^3–6^ the literature on atmospheric exposure routes has only emerged during the past decade.^2,7–13^ Most recently, evidence emerged on atmospheric emissions of known or regulated PFAS compounds from industrial sources,^14–20^ including previously unknown PFAS species, intermediates, or transformation products (products of incomplete combustion, PIC) originating from the incomplete destruction of PFAS during thermal treatment processes.^21–28^ More specifically, besides the 3 main drivers of thermal destruction (turbulence, residence time, and temperature),^22,29^ reaction kinetics demonstrated the synergistic role of oxygen and water vapor in driving the reaction toward HF and CO_2_ (mineralization), while preventing PIC formation.^27,30^ In this regard, it is essential to consider the distinction between thermal destruction; degradation of parent PFAS present in the input material, and mineralization; i.e., complete degradation of parent PFAS, potential intermediates, and transformation products into inorganic fluorine, when evaluating destruction efficiencies. Experimental evidence on atmospheric PFAS emissions is essential to (i) characterize the magnitude and composition of contemporary industrial releases, (ii) better understand the efficiency of thermal destruction technologies, and (iii) create the evidence base for proper mitigation strategies and air quality management. As evidence is growing on the wide occurrence and diversity of PFAS in numerous user products and processes,^31–34^ the waste management sector is challenged to responsibly manage these chemicals at contemporary waste treatment facilities. At full-scale municipal solid waste incinerator (MSWI) plants, PFAS releases have previously been identified in bottom ash, fly ash, scrubber water, flue gas salts, leachate, and flue gases.^15–17,19,35–37^ The first step in responsibly managing these chemicals is adequate monitoring of environmental releases to (i) identify which PFAS species are present in environmental releases, (ii) gain evidence on the impact of full-scale process optimizations (furnace and flue gas abatement), and (iii) allow for environmental impact assessment by translating emissions to ambient air contributions.

Although recent studies documented atmospheric PFAS releases from MSWIs, the resulting ambient air contributions remain unclear. Our study, therefore, aims at (i) characterizing atmospheric PFAS emissions from a contemporary WtE plant under representative operational conditions and (ii) combining stack emission measurements with ambient air sampling to evaluate the resulting emission impact in the vicinity of the WtE plant.

## 2 Materials and Methods

### 2.1 Waste-to-Energy Facility

A municipal solid waste incinerator (MSWI) plant, which in most cases also serves as a waste-to-energy (WtE) plant, incinerates municipal solid waste to produce heat or heat and electricity. Measurements were performed at a WtE plant located in Flanders, Belgium. This facility processes yearly about 140,000 tons of nonrecyclable municipal waste from over 1.1 million people, while recovering energy from the generated heat. The installation can be considered state-of-the-art with a waste bunker to collect the nonrecyclable municipal waste (∼600 tons/day), followed by waste homogenization using rotor shears and cranes, subsequent dosing in 2 grate incinerators at 950 °C (∼9 tons/h with a residence time of ∼45 min), and an extensive flue gas treatment including an electrostatic precipitator (ESP), semiwet scrubber with lime slurry (∼170 °C), bag house filter, and wet scrubber (∼65 °C) before flue gas is released to the atmosphere via the stack (Figure 1). Flue gases are continuously monitored for regulatory pollutants (CO, dust, NOx, SO_2_, HCl, dioxins, NH_3_). Emission measurements were performed under representative incineration and post-treatment conditions.

**Figure 1:**
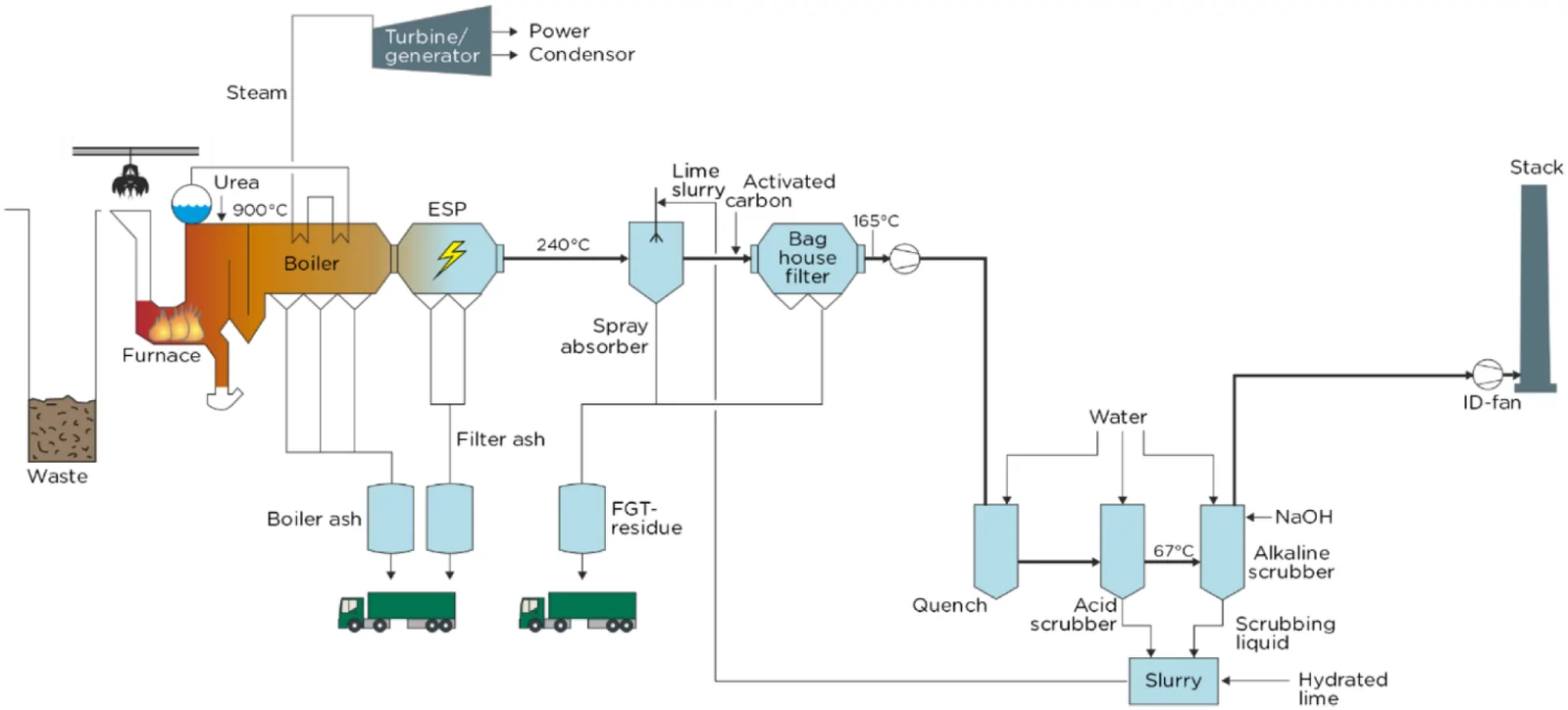
Process flow diagram of the considered waste-to-energy plant.

### 2.2 Monitoring Methods

#### 2.2.1 Emission Monitoring

To date, no ISO or CEN standard exists for the characterization of PFAS in atmospheric emissions, ambient air, or deposition. Nevertheless, a national reference method (LUC/VI/003) was adopted in Belgium, originally published in 2023.^38^ This compendium method is derived from the OTM-45 method published by US EPA^39^ and optimized based on a series of laboratory and field validation experiments.^16,40^ The compendium method describes a sampling and analytical procedure for the quantification of 50 per- and polyfluoroalkyl compounds (PFAS) in a guided gas stream, as the sum concentration in mass per unit gas volume (ng Nm^3–^) of both semivolatile and particle-bound polar PFAS (C4–C18). The sampling procedure entails a minimum of 3 h of isokinetic grid sampling (∼2 N m^3^ of flue gas) with a heated sampling probe and PFAS capture over subsequently a heated quartz fiber filter, polystyrene-divinylbenzene sorbent (XAD-2) module, and impingers filled with ultrapure water (Figure 2). The samples (and rinses) are collected on the stack, transported to the lab where they are aggregated to 6 analytical fractions (filter, water, MeOH and cyclohexane rinse, primary and secondary XAD2), prepared by conducting solvent extraction with sonication (filter and XAD2), solid-phase extraction (SPE; water), and evaporation (MeOH and cyclohexane rinses), and subsequently quantified via ultraperformance liquid chromatography coupled to tandem mass spectrometry (UPLC-MS/MS). More details on the extraction procedure can be found in the LUC/VI/003 standard.^41^ After analysis, the quantified PFAS mass (ng/fraction) is postprocessed by summing all analytical fractions and normalizing for the sampled dry flue gas volume at reference temperature and pressure (Nm^3^) at reference oxygen level (11%), resulting in an emission concentration, expressed as ng PFAS per Nm^3^ of dry flue gas (ng Nm^3–^dr). An analytical compound list with associated quantification limits (LOQ) and measurement uncertainties (MU) is provided in SI1. A number of PFAS compounds can occur in both linear and branched isomers (e.g., PFOA, PFHxS, PFOS, PFOSA, MePFOSA, EtPFOSA, MePFOSAA, and EtPFOSAA). Therefore, a distinction was made between the concentration of the linear form (“L” prefix; e.g., L-PFOS) and the concentration of the combined linear and branched isomers (total, prefix “T,” e.g., T-PFOS). Emission concentrations are reported for every individual PFAS compound and summed as ∑PFAS_42_ (sum of linear (L-) and linear + branched isomeric compounds (T-) when available) and ∑EFSA_4_ (sum of PFHxS, PFOS, PFOA, PFNA) concentrations following the lower-bound principle (<LOQ = 0).

**Figure 2:**
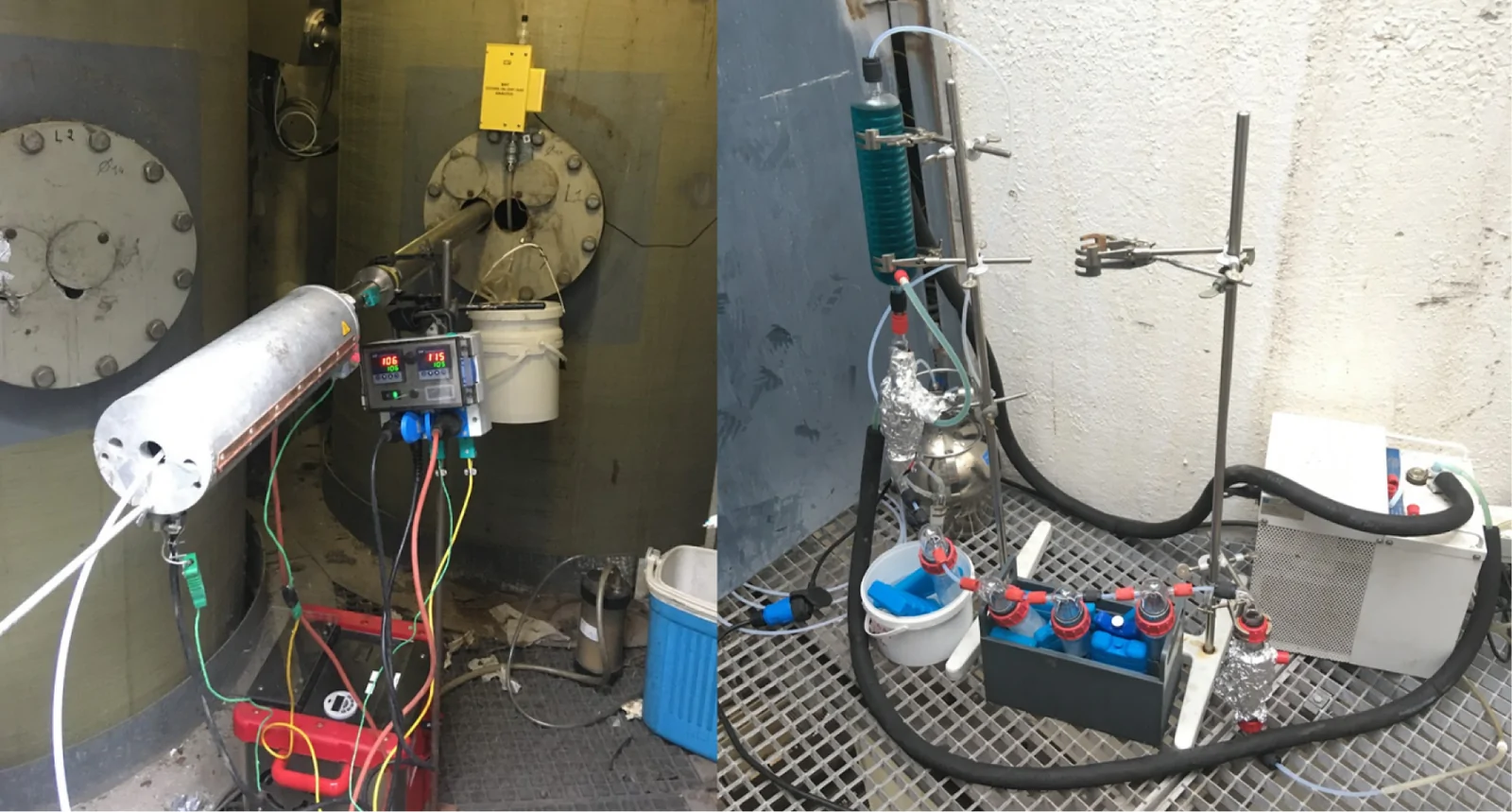
Emission sampling with a heated sampling probe (left) and LUC/VI/003 sampling train including a subsequent condenser, an XAD-2 sorbent, a condensate flask, 3 water-filled impingers, and a secondary XAD-2 sorbent (right).

Besides the 7 field samples, 5 medium-, 6 field-, and 2 postrinse blank samples are collected to evaluate potential background contamination in the applied media, the lab, and the stack environment. Proper sampling is evaluated by means of the postrinse blank, while sampling train breakthrough is evaluated based on a secondary XAD-2 module (criterion: < 30%). For more details on the sampling procedure and performed lab and field validations; see Hofman et al.^16^

#### 2.2.2 Ambient Monitoring

Like emission monitoring, no regulatory reference method is available to date to determine PFAS in ambient air. Therefore, an active air sampling procedure was developed and validated for the same set of 50 semivolatile polar PFAS compounds (C4–C18). Active air sampling was performed at 4 temporary monitoring locations in the vicinity of the waste-to-energy plant using TISCH Environmental high-volume samplers equipped with a total suspended particles (TSP; no size-selective inlet) sampling head containing consecutively a quartz fiber filter (Sartorius grade T293) and precleaned polyurethane foam (PUF) and XAD-4 sandwich (Figure 3). While dust-adsorbed PFAS are captured on the filter, the PUF-XAD-PUF sorbent captures the (semi)­volatile PFAS. The filter and PUF sorbent are sampled continuously for 14 days at a sampling flow of 3.5–4.5 m^3^ h^–1^, collected, and transported to the laboratory where 10 mL (filter) or 450 mL (PUF) of methanol/NH_3_ and internal standard solution (isotopically 13C-labeled PFAS) is added, and extraction by sonication (1 h for the filter, 2 h for the PUF sorbent) is performed. The filter extract is evaporated to 2 mL and the remaining residue removed by centrifugation, while the PUF extract is purified over ENVI-Carb. The extracts are further evaporated to 500 μL and subsequently diluted with 500 μL of ultrapure water. From these extracts, 10 μL is injected and analyzed using UPLC-MS/MS. The sum of the quantified PFAS on the filter and PUF (ng/filter or ng/PUF per analyzed PFAS compound) is normalized for the sampled air volume (m^3^) to obtain the ambient PFAS concentration, expressed as ng m^–3^. Ambient PFAS concentrations are reported for every individual PFAS compound and summed over the entire PFAS analytical scope (∑PFAS_42_) and ∑EFSA_4_ (sum of PFHxS, PFOS, PFOA, PFNA) concentration following the lower bound principle (<LOQ = 0). In addition to the field sample collection, procedure blanks are considered for every analytical series (blank filter and PUF that are prepared together with the field samples) and field blanks of filter and PUF samples that are prepared and treated similarly and stored on-site during sampling.

**Figure 3:**
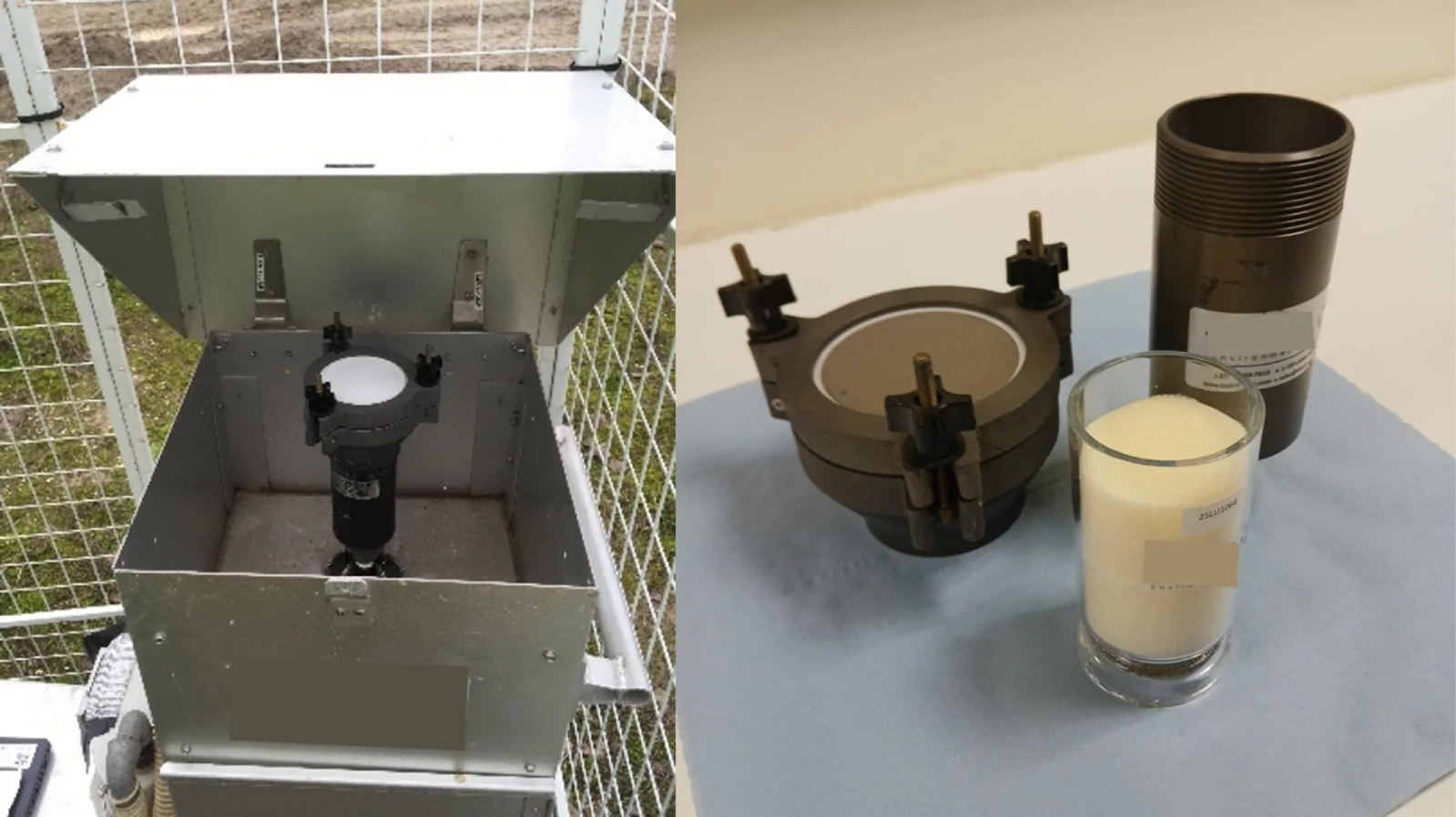
Ambient air sampler (left) with the sampled filter and PUF-XAD-PUF sandwich (right).

Although no monitoring standard exists to date, this ambient monitoring approach is comparable to other exploratory studies combining filter and PUF sorbents with active air sampling^42,43^ and was extensively validated and applied before to evaluate the ambient PFAS concentration and composition during 2022 and 2023 at 16 ambient monitoring locations over Flanders, Belgium.^44,45^

The considered PFAS compounds, their associated measurement uncertainty U (k = 2), and recovery based on a validation experiment are provided in SI2. The quantification limit (LOQ) was determined separately for the filter and PUF and is on average 0.0004 ng m^–3^ for the filter and 0.0011 ng m^–3^ for the PUF. Most of the individual PFAS compounds are analyzed with a LOQ of <0.001 ng m^–3^, which is significantly lower than the temporary ambient air assessment framework of 0.3 ng m^–3^ for ∑EFSA4 (sum of PFHxS, PFOS, PFOA, PFNA) considered in Flanders, Belgium.^44,46^

### 2.3 Monitoring Plan

This study reports on 7 emission measurements conducted at the stacks of 2 grate incinerators (lines 1 and 2) of the WtE plant. The emission campaigns were conducted in 2023, on, respectively, April 14th, May 4th, August 23rd, and October 12th, under representative and constant operational conditions. While the first 3 campaigns (EM1–EM3) involved simultaneous emission measurements on both grate incinerators (X_L1_ and X_L2_ in Figure 4), EM4 only involved line 1 to confirm the impact from a process optimization. The 4 emission campaigns (EM1–EM4) resulted in 7 individual stack measurements (3 replicates on line 2 and 4 replicates on line 1), reported in ng Nm^3–^ of flue gas, respectively, for each individual PFAS compound, the sum of the 42 considered target compounds (∑PFAS_42_), and the sum of T-PFHxS, T-PFOS, T-PFOA, and PFNA (∑EFSA4).

**Figure 4:**
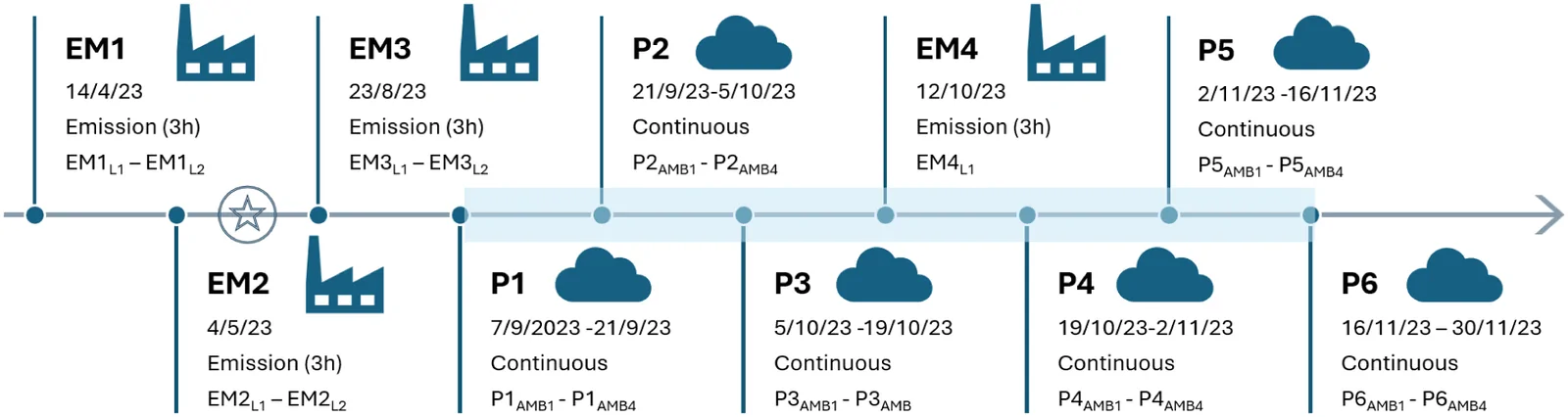
Applied monitoring strategy combining 4 emission campaigns (EM1–EM4), each consisting of 3 h of flue gas sampling at both lines (X_L1_ and X_L2_) for EM1, EM2, and EM3 and 3 h of flue gas sampling at line 1 during EM4 (EM4_L1_), with continuous ambient air monitoring at 4 sites (X_AMB1_–X_AMB4_) between 7/9/2023 and 30/11/2023 (shaded area), subdivided in 6 sequential sampling periods (P1–P6). The AC dosage optimization between EM2 and EM3 is represented on the timeline by the star.

Besides stack emission monitoring, an ambient monitoring campaign was conducted for nearly three months (12 weeks; P1–P6) at 4 monitoring sites (X_AMB1_–X_AMB4_) in the vicinity of the WtE plant. The monitoring sites AMB1, -3, and -4 were positioned assuming a predominant southwesterly wind direction, respectively, 900 m upwind and 450–1250 m downwind of the WtE plant (Figure 4). AMB2 is located 600 m SE of the plant. Ambient monitoring resulted in 6 consecutive ambient air replicates (sum of the filter and PUF) at each of the 4 monitoring sites (Table 1), reported in ng m^–3^, respectively, for each individual PFAS compound, the sum of the 42 considered target compounds (∑PFAS_42_), and the sum of T-PFHxS, T-PFOS, T-PFOA, and PFNA (∑EFSA_4_). Ambient monitoring data was lost for one sampling period (weeks 9–10) at the AMB1 site due to a blown-over air sampler.

**Table 1:** Meteorological Conditions Measured at a Nearby Monitoring Station from the Belgian Royal Meteorological Institute during the Respective Ambient Air Sampling Periods (P1–P6)

**MONITORING PERIOD**			**TEMP**	**PRECIPITATION**	**WIND DIRECTION (%)**				**WIND VELOCITY**
START	STOP	PERIOD	°C	mm	N–E	E–S	S–W	W–N	m s^–1^
07/09/2023	21/09/2023	P1	19.0	14.6	21%	34%	29%	10%	2.7
21/09/2023	05/10/2023	P2	15.5	0.4	0%	37%	52%	7%	3.0
05/10/2023	19/10/2023	P3	14.0	22.6	15%	14%	63%	4%	3.6
19/10/2023	02/11/2023	P4	11.5	54.4	9%	34%	53%	3%	4.3
02/11/2023	16/11/2023	P5	8.8	47.6	3%	13%	76%	4%	4.7
16/11/2023	30/11/2023	P6	6.7	29.6	7%	10%	56%	21%	4.0

To our knowledge, no widely accepted emission limit values (ELVs) or legal assessment frameworks exist to date. In The Netherlands, general emission limits are defined for substances of very high concern (SVHC), among others 11 PFAS, of 0.05 mg/Nm^3^ (MVP1; aerosols) and 1 mg/Nm^3^ (MVP2; gases).^47^ In the US, a few state regulations vary significantly in terms of form (e.g., guidance documents, screening levels, and/or ambient air limits), established ambient concentration limits (0.0001–10 μg m^–3^), or PFAS compounds considered.^48,49^ As no ELVs are currently available, emissions and ambient concentration measurements were benchmarked against the temporary assessment framework for ambient air in Flanders, Belgium.^46^ This assessment framework is derived from the EFSA tolerable weekly intake (TWI) of 4.4 ng kg^–1^ body weight per week, defined in 2020,^50^ and can be considered an inhalation equivalent by assuming REACH guidelines on inhalation volume (20 m^3^ day^–1^), average body weight (70 kg), and an allocation factor of 20%.^51^ This temporary assessment framework in Flanders, Belgium, was originally defined as 0.4 ng m^–3^ for the chronic (yearly averaged) exposure to the sum of PFHxS, PFOS, PFOA, and PFNA by Peters et al.^52^ and later strengthened to 0.3 ng m^–3^ and extended with additional compound-specific limit values for HFPO-DA (1.4 ng m^–3^), 6:2FTS (30 ng m^–3^), PFBS (140 ng m^–3^), PFPrA (245 ng m^–3^), and PFBA (460 ng m^–3^).^46^ When translating the proposed ambient air limits of the temporary assessment framework to stack emission concentrations for varying stack types (height and heat content) by means of inverse modeling,^49^ the resulting emission limits (0.0164–50.67 kg/j in Vander Aa et al.^49^) are much more stringent than the ELV proposed in The Netherlands (17.5–350 kg j^–1^ considering 40,000 m^3^ h^–1^) or the US (1–10000 ng m^–3^ for individual PFAS vs 0.3 ng m^–3^ for ∑EFSA_4_). The most stringent (Belgian) assessment framework was, therefore, used to benchmark ambient air and emissions against.

Meteorological conditions during the ambient air sampling period were derived from a nearby meteorological station of the Belgian Royal Meteorological Institute (RMI; https://opendata.meteo.be/), including atmospheric temperature (°C), precipitation (mm), wind direction (%), and wind speed (m s^–1^).

## 3 Results and Discussion

### 3.1 Meteorological Conditions

For each consecutive 14-day ambient monitoring period (AMB1–AMB6), atmospheric temperature (°C), precipitation (mm), wind direction (% per wind sector), and wind velocity (m s^–1^) are summarized in Table 1. A total precipitation of 169 mm was measured during the monitoring campaign. Precipitation was much lower during the first half of the monitoring campaign (September–October 12th), compared to the second half (>October 12th), as shown in SI3.

During the monitoring campaign, a prevailing wind direction in the 180–270° segment (54% of the time) was observed (Figure 5), followed by the 90–180° segment (24%), 0–90° segment (10%), and 270–360° segment (8%). The prevailing southwesterly wind direction confirms the representative positioning of the ambient monitoring locations up- (AMB1) and downwind (AMB3 and AMB4) of the WtE plant (Figure 8). The exhibited wind distributions for each of the consecutive sampling periods (P1–P6) in Table 1 can be found in SI4.

**Figure 5:**
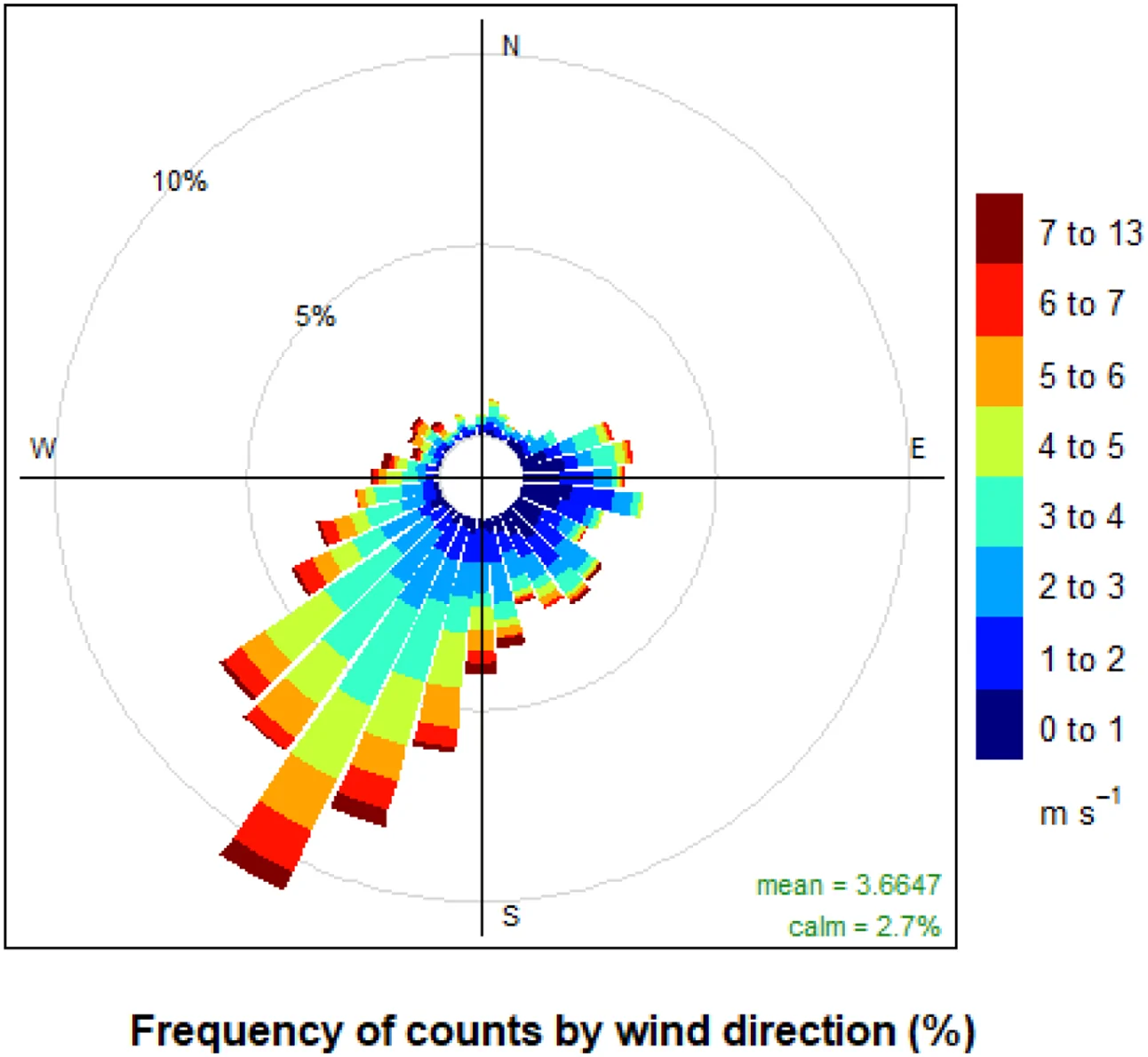
Observed wind distribution during the ambient monitoring campaign. Wind distributions for each individual sampling period (P1–P6) can be found in SI4.

### 3.2 MSWI Emissions

#### 3.2.1 Emission Measurements

Quantified emission concentrations (ng Nm^3–^dr) for each emission campaign are provided in SI5 and show varying emission concentrations respective incineration lines (L1-L2) and emission campaigns (EM1-EM4), ranging from 133 to 4049 ng Nm^3–^dr for ∑PFAS_42_ and 7 to 357 ng Nm^3–^dr for ∑EFSA_4_. The first 2 measurement campaigns (EM1 and EM2) showed pronounced concentration differences between both incinerator lines (on average 3108 ng Nm^3–^dr vs 156 ng Nm^3–^dr), as illustrated in Figure 6. After evaluating operating conditions between both emission lines, a lower dosage of activated carbon in the flue gas treatment of line 1 was discovered. After optimizing the activated carbon dosage of line 1 to 5 kg hour^–1^ (similar to line 2), new emission measurements (EM3) showed significant emission reductions (206 ng Nm^3–^dr) on line 1, down to the concentration level of line 2 (255 ng Nm^3–^dr). A final emission measurement on line 1 in October (EM4) yielded 133 ng Nm^3–^dr confirming the observed trend (Figure 6). As a result of the 90-95% emission reduction at line 1, several PFAS compounds approach field blank levels, resulting in exceedances of the field blank criterion (emission sample <10× field blank concentration) in EM3 and EM4. An extensive evaluation of all LUC/VI/003 quality controls (QCs) including sampling standard (SS) recovery (%), sampling train breakthrough (%), and medium blank levels is provided in SI6.

**Figure 6:**
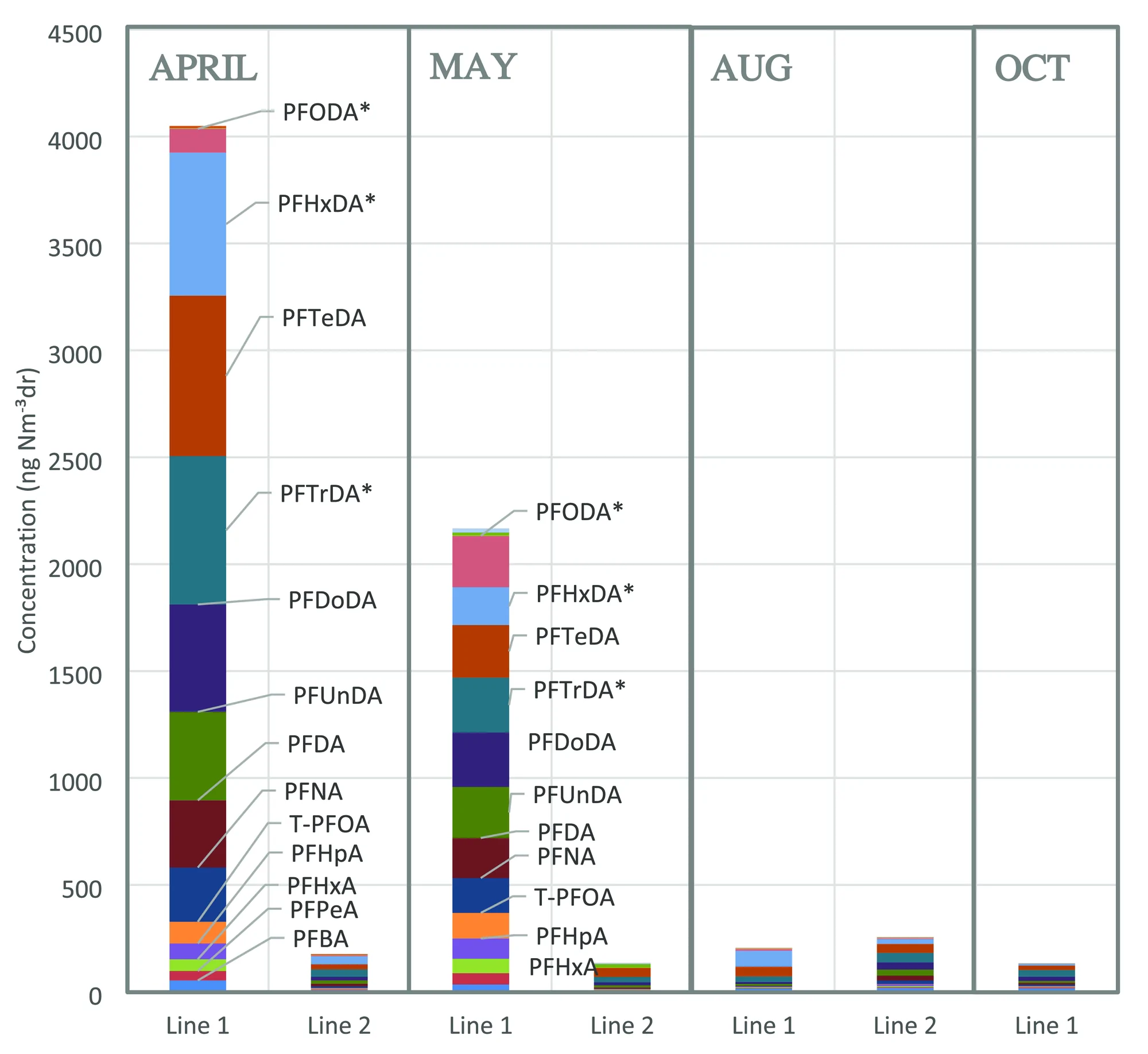
Quantified ∑PFAS_42_ emission concentration (ng Nm^3–^dr) and composition at incinerator lines 1 and 2 in April (EM1), May (EM2), August (EM3), and October 2023 (EM4). * indicated compounds exhibiting higher (>50%) measurement uncertainty.^53^.

When evaluating the PFAS composition between the different campaigns and incineration lines (Figure 6), a rather stable fingerprint can be observed with almost exclusively carboxylic acids (PFCAs) ranging from C4 (PFBA) to C18 (PFODA) and dominated (>75%) by long-chain compounds (C8–C18; PFNA–PFODA). The selectivity of the dosed activated carbon for larger molecules is suggested, as the relative contribution of PFBA on line 1 increases from 1 to 2% to 7–13% after increasing the AC dosage (Figure 7).

**Figure 7:**
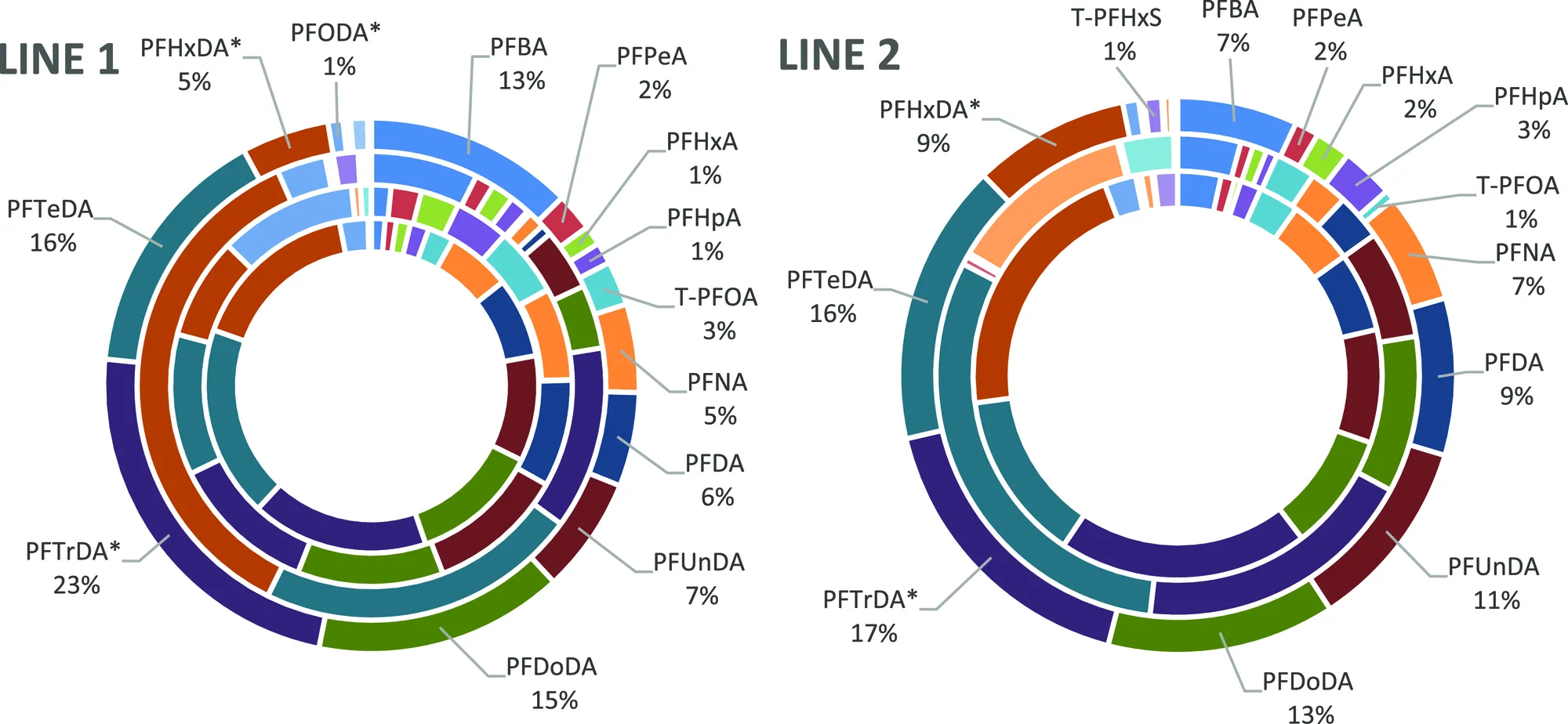
Percentage contribution (%) of individual PFAS compounds to the ∑PFAS_42_ sum for each emission measurement (inner/older vs outer/most recent rings) at incineration lines 1 (left) and 2 (right).

Field emission data from full-scale operating facilities is still scarce.^22^ Hofman et al.^16^ reports on 15 repeated stack emission measurements with the same standard as applied in our study (LUC/VI/003) at 3 hazardous waste incinerators (rotary kilns) in Belgium, resulting in 47–1987 ng Nm^3–^dr for ∑PFAS_42_ (predominantly PFCAs) and 10–509 ng Nm^3–^dr for ∑EFSA_4_, and factor >10 reduction in emissions over time due to flue gas treatment optimizations in the scrubber and activated carbon filters.^16^ In the US, flue gas monitoring using EPA Method 10 adjusted for PFAS at a sewage sludge incinerator (SSI) resulted in semivolatile ∑PFAS_30_ concentrations of 523 ng Nm^3–^ dominated by HFPO-DA (488 ng Nm^3–^), PFCAs with PFBA up to PFTeDA (total of 27.3 ng Nm^3–^), 6:2FTS (1.96 ng Nm^3–^), and 8:2FTS (1.3 ng Nm^3–^).^54^ Strandberg et al.^36^ evaluated collected residual samples from 27 incineration plants in Sweden and detected PFAS in bottom ash (0.22–13 μg kg^–1^), fly ash (0.18–38 μg kg^–1^), and condensate water (0.3–183 ng L^–1^), with predominantly PFCAs and a large representation of short-chain PFCAs. No apparent relationship was found between the analyzed concentrations of PFAS in the sampled matrices and the operational data. In a follow-up study, Strandberg et al.^17^ applied 2 methods for semivolatile polar PFAS, namely, a modified cooled probe method based on EN 1948:1 and OTM-45 and additionally OTM-50 for volatile nonpolar PFAS under real operating conditions at four Swedish WtE plants (3 grate furnaces and 1 rotary kiln). Volatile nonpolar (OTM-50) results yielded ∑PFAS_30_ between 110 and 4605 μg Nm^3–^ with mainly hydrofluorocarbons (HFCs) and perfluorocarbons (PFCs), while OTM-45 and EN 1948:1 detected ∑PFAS_54_ between 0.33 and 39 ng Nm^3–^ dominated by short-chain (<C8) PFCAs and 6:2FTS.^17^ Björklund et al.^15^ evaluated 18 polar semivolatile target PFAS in ashes, process water, and flue gases from a municipal solid waste incinerator (MSWI) in Sweden. They identified PFAS in all examined residues, with short-chain PFCAs being the most abundant, but much lower flue gas concentrations in the order of 3.4–5.6 ng m^–3^, collected with the modified EN 1948:1 (cooled probe) method.^15^ More recently, thermal degradation was evaluated at a Swedish WtE plant by characterizing flue gases and residual emissions (bottom and fly ash, gypsum, water, and sludge) for PFAS.^19^ Flue gases were evaluated at the electrostatic precipitator (before flue gas treatment) and at the stack for 71 polar semivolatile (OTM-45) and 28 nonpolar volatile (OTM-50) compounds. The flue gas semivolatile polar fingerprint was dominated by carboxylic acids (PFCAs) including PFBA, PFPrA, PFPeA, and PFOA (total 14.96 ng Nm^3–^), lower concentrations of sulfonic acids (PFSAs) including PFOS and PFHxS (total 1.69 ng Nm^3–^) and 6:2FTS (1.69 ng Nm^3–^). Volatile nonpolar PFAS were several orders of magnitude higher and dominated by hexafluoropropene (C_3_F_6_) at 1500 μg Nm^3–^ and chlorodifluoromethane (CHClF_2_) at 120 μg Nm^3–^. Volatile nonpolar compounds showed similar concentration levels before and after flue gas treatment, while semivolatile PFAS concentrations decreased to 2.5–5 ng Nm^3–^, with a notable PFBA transfer toward the liquid phase (HCl scrubber). Several semivolatile and volatile PFAS were detected as well in the field blanks.^19^ Although available field studies report a wide range of flue gas concentrations (<1–1987 ng Nm^3–^) which can be explained by the current lack of standardized sampling methods and deviating analytical scopes (18–72 target PFAS), a predominance of PFCAs in flue gases and quantifiable impacts from flue gas treatment systems seem systematic across the cited field studies and substantiate the findings of our study. Nonetheless, ongoing national and international standardization efforts (e.g., CEN TC264 WG48) are instrumental to obtain robust and comparable emission results and facilitate responsible PFAS management in the years to come.

#### 3.2.2 Emission Modeling

The impact of the quantified stack emissions is modeled using the Immission Frequency Distribution Model (IFDM), a bi-Gaussian model that is widely used for environmental impact assessments (EIAs) in Flanders, Belgium.^55^ The model is applied without the building downwash module, as high stacks (60 m) are considered in this study. The model simulates a full year (meteorology from 2017), assuming constant emissions. The annual average concentration is modeled based on the sum of the average mass flow rates (emission concentration × volume flow rate) on both incinerator lines. The maximum daily average concentration is calculated by modeling the sum of the maximum mass flow rates (worst-case) of each line for a full year and subsequently determining the maximum daily average concentration, i.e., the day with the least favorable dispersion and dilution conditions.

Considering the fluctuating emission concentration levels over time at incinerator line 1, emissions are modeled according to three emission scenarios:

• **Scenario 1**: Annual average and maximum daily concentrations **before process optimization** on line 1: based on the sum of the average/maximum of two measurements before optimization (April and May) on line 1 and the average/maximum of all measurements (April, May, and August) on line 2.

• **Scenario 2**: Annual average and maximum daily concentrations based on **all measurements**: based on the sum of the average/maximum of all measurements on line 1 and the average/maximum of all measurements on line 2.

• **Scenario 3**: Annual average and maximum daily concentrations **after process optimization** on line 1: based on the sum of the average/maximum of two measurements after optimization (August and October) on line 1 and the average/maximum of all measurements (April, May, and August) on line 2.

Calculated mass flow rates (mg h^–1^) for each of the three scenarios are provided in SI7. Considering the average/maximum for all emission measurements (scenario 2), the mass flow rates amount to 78/173 mg h^–1^ for ∑PFAS_42_ and 8/15 mg h^–1^ for ∑EFSA_4_. Other stack characteristics applied during scenario modeling are provided in SI7. No ambient background contribution of PFAS is considered in the model, although we know that many sources contribute to urban and rural background concentration levels in Flanders.^44,45^

A map of the modeled annual average ∑PFAS_42_ contribution from the WtE plant following model scenario 3 (after process optimization) is provided in Figure 7. Due to the stack effect, the lowest ambient concentrations are found near the stack and higher concentrations slightly further downwind (northeast) of the emission source, close to the AMB3 monitoring location (Figure 7). From the modeled concentration variability around the stack, the maximum concentration level within the model domain is provided for each of the considered model scenarios in SI8. Generally, low concentrations are observed, with an annual average contribution of 0.025 ng m^–3^ (before optimization; scenario 1), 0.014 ng m^–3^ (all measurements; scenario 2), and 0.0027 ng m^–3^ (after optimization; scenario 3) for ∑PFAS_42_. For ∑EFSA_4_, modeled annual average contributions amount to 0.003 ng m^–3^ (before optimization; scenario 1), 0.0014 ng m^–3^ (all measurements; scenario 2), and 0.0002 ng m^–3^ (after optimization; scenario 3). Modeled long-term (yearly averaged) source contributions (ng m^–3^) fall well below the temporary assessment framework (0.3 ng m^–3^) for ∑EFSA_4_ (0.0002–0.003 ng m^–3^; 2000–133× below the limit value). When focusing on the meteorological conditions of the considered ambient monitoring period (7/9–30/11), scenario 3 results in maximum atmospheric contributions of 0.00283 ng m^–3^ for and 0.000236 ng m^–3^ for ∑EFSA_4_ within the considered model domain (Figure 8).

**Figure 8:**
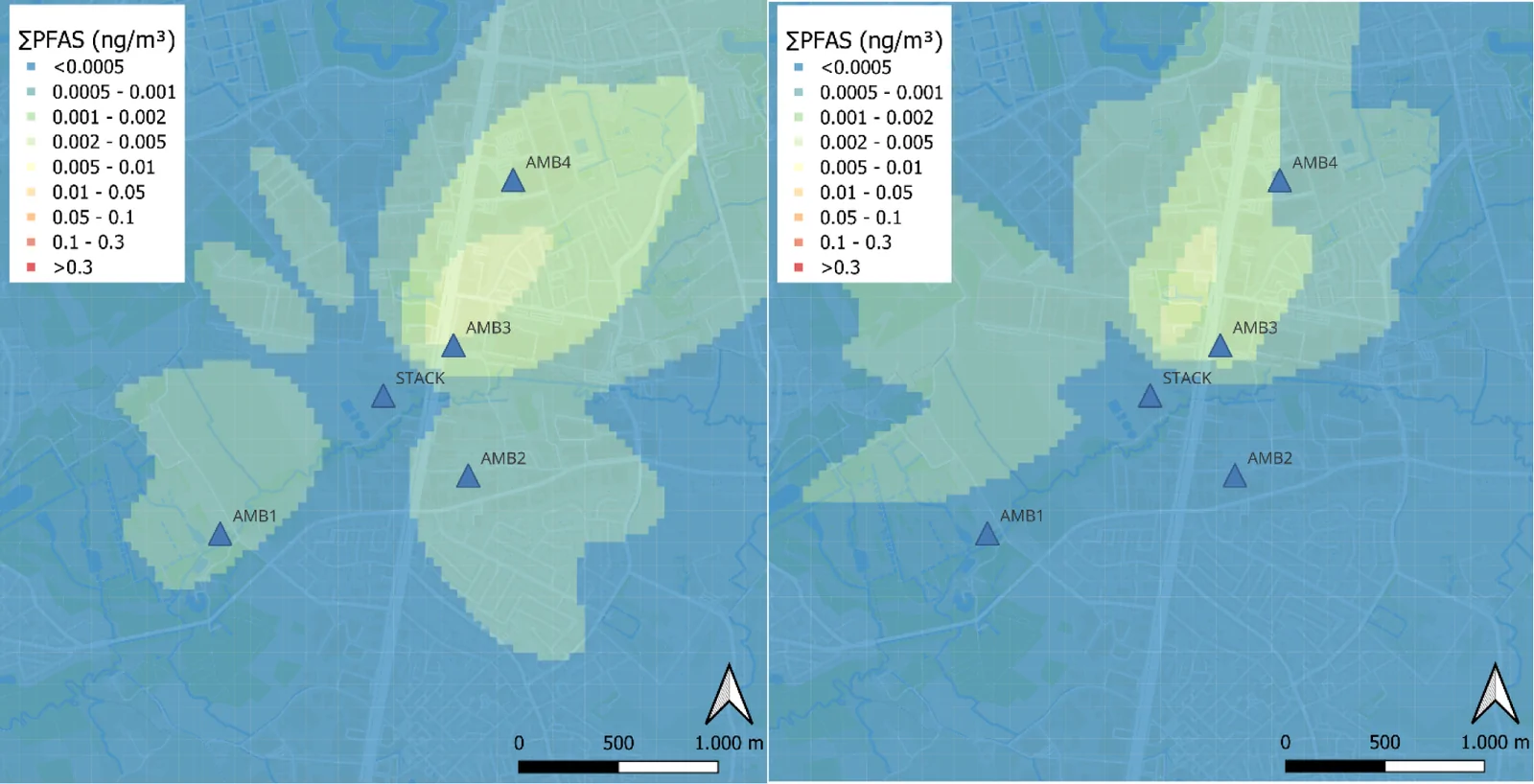
Map of the modeled annual average ∑PFAS_42_ contribution in the vicinity of the WtE plant, considering stack emissions after process optimization (scenario 3) during a full year (2017) of meteorological conditions (left) and the experienced meteorological conditions during the ambient monitoring campaign (right).

### 3.3 Ambient PFAS Concentration

Field blank concentrations of filters (6) and PUFs (6) were all below the quantification limit (LOQ), except for one PFAS on the filter (T-PFOA) and four PFAS on the PUF (L-/T-PFOA, 6:2diPAP, and ADONA: 0.001–0.024 ng m^–3^). It can be concluded that generally, no quantifiable concentrations, and in exceptional cases very low concentrations, are measured in the field blanks. Therefore, no contamination or other background effects need to be considered in the further evaluation of the measurement results.

Average ambient PFAS concentrations at the considered monitoring locations range from 0.022 (AMB1) to 0.073 ng m^–3^ (AMB3) for ∑PFAS_42_ and 0.003 (AMB1) to 0.023 ng m^–3^ (AMB3) for ∑EFSA_4_, which are significantly lower than the limit values defined in the temporary assessment framework for ambient air in Flanders, Belgium.^46^ An overview of the measured concentration levels for the consecutive campaigns (P1–P6) is provided in Table 2.

**Table 2:** Measured ∑PFAS_42_ Concentrations (ng m^–3^) for Each of the Sampling Periods (P1–P6) and Considered Monitoring Locations (AMB1–AMB4) with Associated Wind Contribution (%) in Each Quadrant

	Ambient ∑PFAS_42_ (ng m^–3^)				Wind direction (% in quadrant)			
Period	AMB1	AMB2	AMB3	AMB4	N–E	E–S	S–W	W–N
**P1**	0.03	0.05	0.10	0.04	21%	34%	29%	10%
**P2**	0.03	0.03	0.07	0.03	0%	37%	52%	7%
**P3**	**0.02**	**0.03**	**0.06**	**0.03**	**15%**	**14%**	**63%**	**4%**
**P4**	0.08	0.01	0.07	0.02	9%	34%	53%	3%
**P5**	-	0.02	0.08	0.03	3%	13%	76%	4%
**P6**	0.02	0.02	0.04	0.03	7%	10%	56%	21%
*AVG*	***0.035***	***0.025***	***0.069***	***0.030***	***9%***	***24%***	***55%***	***8%***
*MEDIAN*	***0.032***	***0.022***	***0.069***	***0.028***	***8%***	***24%***	***55%***	***6%***

None of the limit values for ∑EFSA_4_ (0.3 ng m^–3^), HFPO-DA (1.4 ng m^–3^), 6:2FTS (30 ng m^–3^), PFBS (140 ng m^–3^), PFPrA (245 ng m^–3^), and PFBA (460 ng m^–3^) were exceeded during this study. Moreover, the monitoring location which exhibits the highest ∑EFSA_4_ concentration (AMB3) is still 13× lower than the temporary assessment framework of 0.3 ng m^–3^.

Location AMB3, the nearest and downwind of the waste-to-energy plant, exhibits higher total ∑PFAS_42_ and ∑EFSA_4_ concentrations compared to the other ambient monitoring locations, and a distinct PFAS fingerprint with long-chain carboxylic acid (PFCA) contributions from C9 up to C16 (PFNA, PFDA, PFUnDA, PFDoDA, PFTrDA, PFTeDA, PFHxDA), in addition to the more commonly detected PFAS compounds at the other locations, e.g., carboxylic acids (PFBA, PFHxA, PFHpA, PFOA), sulfonic acids (PFBS, PFOS), and sulfonamides (PFOSA, PFBSA), as illustrated in Figure 8.

Perfluorocarboxylic acids (PFCAs) and perfluorosulfonic acids (PFSAs) make up most of the ambient PFAS fingerprint, respectively, 51–91% and 7–13% of ∑PFAS_42_, with notable location differences in short- vs long-chain PFCA contribution (Table 3). While short-chain (C4–C7) PFCAs dominate (42–71% of ∑PFAS_42_) at 3 of the 4 locations (AMB1, -2, and -4), the nearest downwind location AMB3 exhibits predominantly long-chain (≥C8) PFCAs (60%).

**Table 3:** Relative Contribution (%) of Short- and Long-Chain PFCAs and PFSAs to the Ambient ∑PFAS_42_ Concentration at Each of the Considered Ambient Air Monitoring Locations

	PFCAs		PFSAs	
STATION	C4–C7	≥C8	C4–C5	≥C6
AMB1	42%	9%	2%	8%
AMB2	69%	11%	3%	10%
AMB3	31%	60%	1%	6%
AMB4	71%	13%	2%	10%

Temporal concentration variability at the considered ambient monitoring locations ranges between 0.01 and 0.1 ng m^–3^for ∑PFAS_42_ and 0.002 and 0.05 ng m^–3^ for ∑EFSA_4_ (SI9). The temporary assessment framework proposed by Vanhooren et al.^46^ is never exceeded; higher concentrations are consistently observed at AMB3 with overall decreasing concentrations during the second half of the monitoring campaign, possibly related to the increasing precipitation (Table 1). The observed concentration increase at AMB3 of ∼0.04/0.017 ng m^–3^ for ∑PFAS_42_/∑EFSA_4_ compared to the other ambient monitoring locations is, respectively, 14/71× higher than the yearly averaged model estimates of 0.0028/0.00024 ng m^–3^ for ∑PFAS_42_/∑EFSA_4_ and 2/9× higher than the maximum daily average model estimates of 0.02/0.002 ng m^–3^ for ∑PFAS_42_/∑EFSA_4_ under model scenario 3 (SI8). Potential explanations can be found in the lack of a simultaneous measurement at line 2, model parametrization and assumptions (e.g., meteorology of 2017), lack of overlap between emission and ambient monitoring campaigns while the presence of other contributing atmospheric PFAS sources in the vicinity of AMB3 cannot be excluded. To evaluate the impact from meteorology, the model simulations were repeated with meteorological data of the considered ambient monitoring period (7/9–30/11), resulting in comparable source contribution maps with ∑PFAS_42_ contributions of 0.001–0.002 ng m^–3^ at AMB3 (Figure 8). As no deposition flux is considered by the model at the considered (short) distance to the stack, the observed deviation can only be explained by model representation of dilution and postcombustion reactions or additional source contributions near the WtE plant. Atmospheric PFAS modeling is a new research field with a lot of unknowns to date, e.g., compound-specific partitioning in atmospheric volatile, water-soluble, or dust-size fractions and inertness and/or reactivity of the compounds in the flue gas. Future studies should, therefore, focus on model validation near isolated point sources, compound-specific partitioning in wet/dry aerosol or volatile atmospheric fractions, and impacts of dry and wet deposition fluxes.

Studies reporting on ambient PFAS concentrations are scarce Figure 9. Peters et al.^44^ report on ambient PFAS levels at 16 different (background to hotspot) locations over Flanders, Belgium, with concentrations ranging from 0.09 to 3.3 ng m^–3^ for ∑PFAS_42_ and 0.001 to 1.319 ng m^–3^ for ∑EFSA_4_ and the highest contributions from PFPrA, PFBA, PFOA, PFOS, and MePFBSA. Ambient air results of our study are within the observed concentration range of Peters et al.^44^ and confirm predominant PFSA and PFCA contributions. Reis et al.^45^ report complementary nontarget results for 51 ambient air and deposition samples indicating that only 13% of detected ionizable PFAS signals correspond to conventional target PFAS, understating the need to further expand the analytical coverage of future monitoring programs.

**Figure 9:**
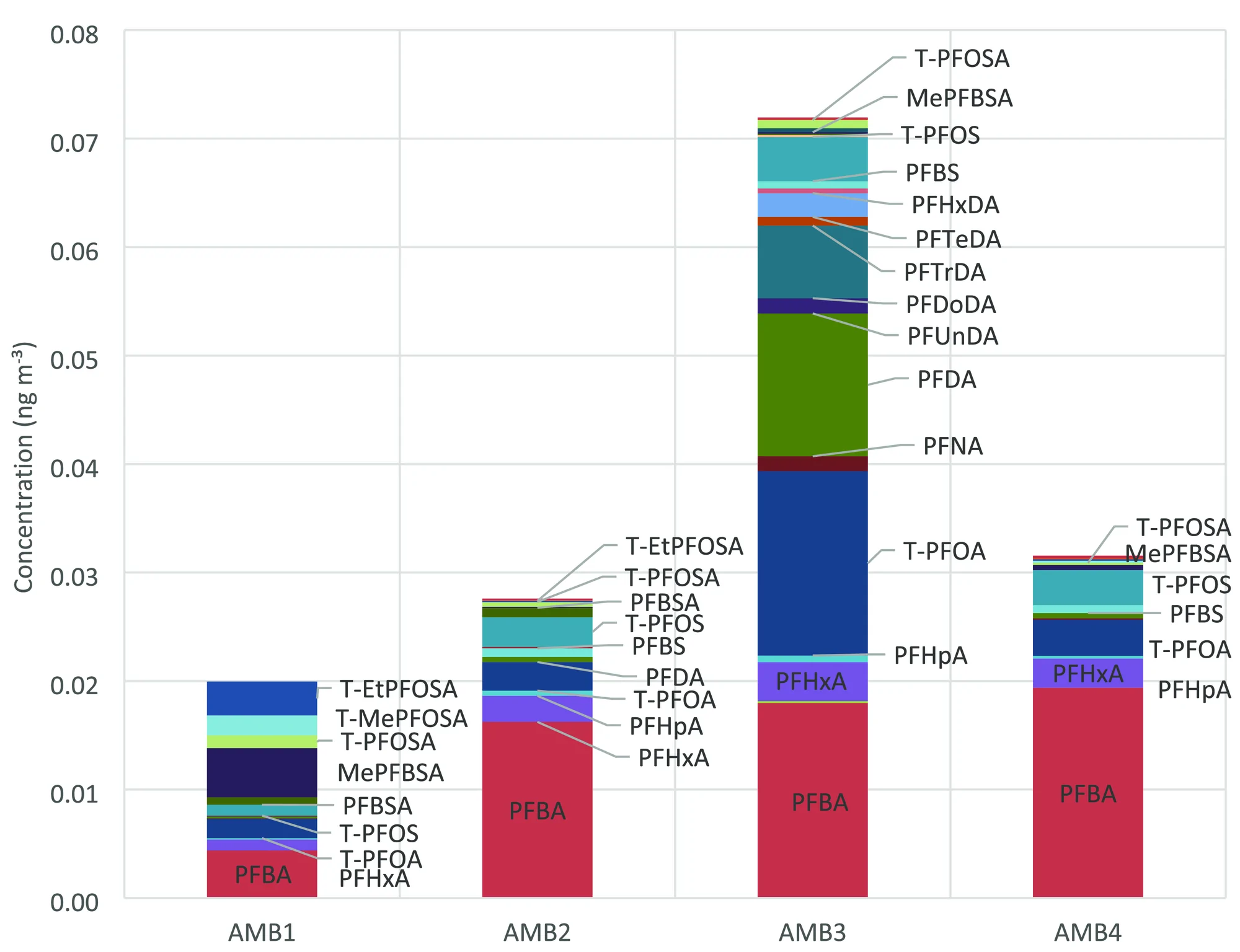
Difference in average ∑PFAS_42_ concentration (ng m^–3^) and composition between the considered ambient monitoring locations (AMB1–4).

### 3.4 Emission Impact of the WtE Plant

To our knowledge, this study is the first to experimentally evaluate the emission impact of a contemporary WtE plant by combining stack emission measurements of 42 semivolatile PFAS with ambient air measurements in the surroundings. We quantified PFAS in the flue gas of 2 grate incinerator stacks under representative operational conditions to evaluate composition and concentration levels of the emissions and assess the resulting emission impact at ambient monitoring locations in the vicinity of the WtE plant. Although emission and ambient air measurements were not synchronized fully (Figure 4), the obtained emission data, model results, and exhibited meteorological conditions (wind direction) substantiate the observed ambient concentration levels. More specifically, the emission measurement from October (EM4) was performed during the third ambient monitoring period (P3 in Table 3, in bold), when exhibited wind distribution was 63% of the time in the S–W quadrant (Table 3). Although no simultaneous emission measurement was performed on line 2, we can consider the 3 previous measurements to be representative for the MSWI emissions at line 2 as (i) both concentration level (Figure 5) and composition (Figure 6) remained constant between the conducted emission campaigns on line 2 and (ii) operating conditions of the incinerator, postcombustion chamber (PCC), and flue gas treatment system remained unchanged according to the plant operators.

Considering the prevailing southwesterly wind direction (55% of the time), monitoring locations AMB3 and AMB4 are predominantly positioned downwind of the WtE plant, respectively, at 450 and 1250 m from the incinerator stack. A decreasing PFAS ambient air concentration is observed with increasing distance to the WtE plant, from 0.069 ng m^–3^ at AMB3 to 0.03 ng m^–3^ at AMB4. Under the exhibited meteorological conditions of the 3-month ambient monitoring campaign, systematically higher (on average 2.3-fold) ∑PFAS_42_ concentrations are observed at location AMB3 (0.069 ng m^–3^), compared to the 3 other ambient monitoring locations (0.025–0.035 ng m^–3^) (Figure 10). Moreover, the PFAS composition at AMB3 (Figure 9) reflects the emission fingerprint dominated by long-chain PFCAs, as observed at emission lines 1 and 2 (Figure 6). At 1250 m downwind of the WtE plant (AMB4), the PFAS concentration (∑PFAS_42_ = 0.033 ng m^–3^) falls back to similar levels as observed at upwind ambient monitoring locations (AMB1 and AMB2), representative of PFAS background levels^44^ and/or other source contributions in the vicinity of the WtE plant.

**Figure 10:**
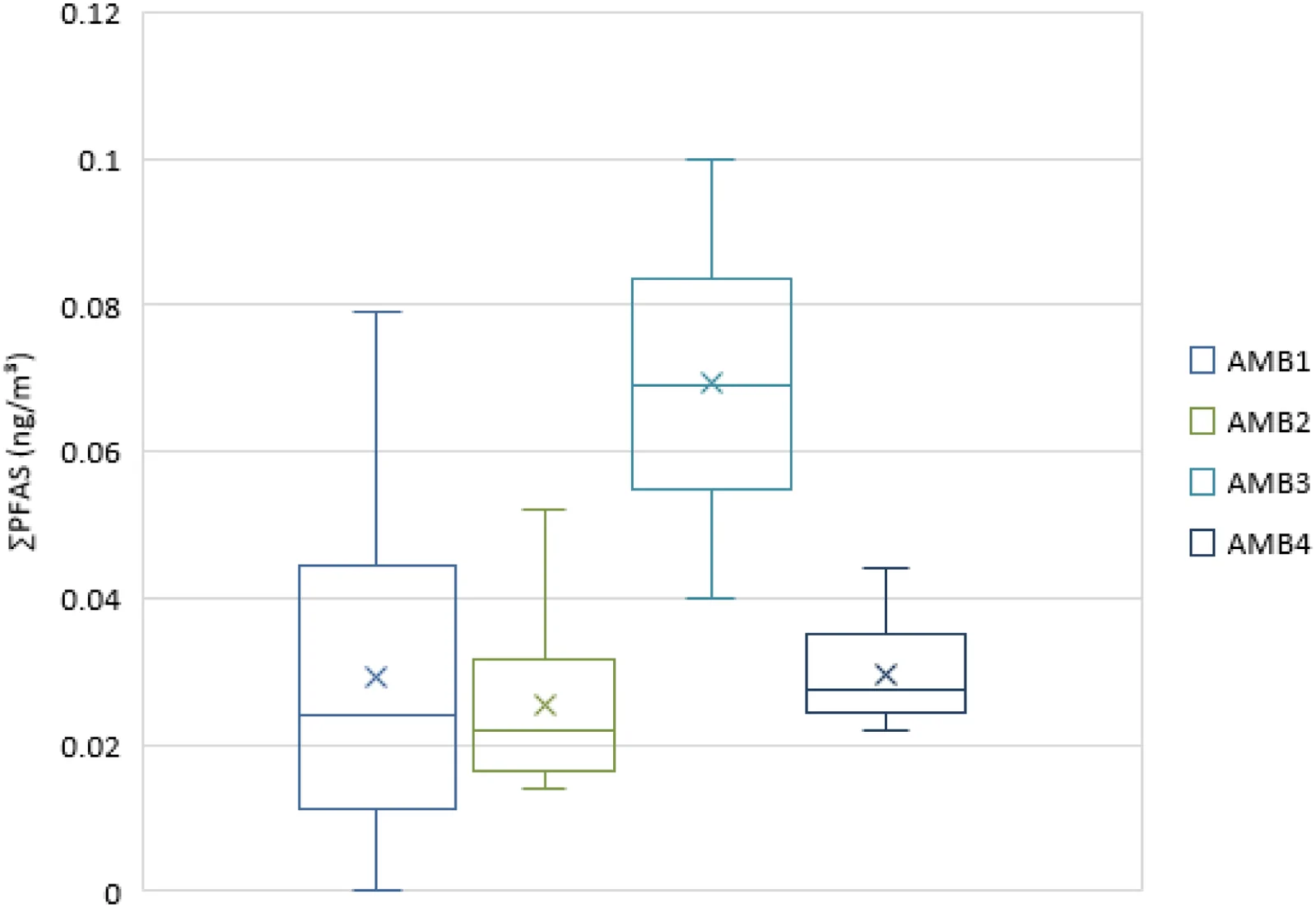
Boxplots representing the measured ambient ∑PFAS_42_ variability (ng m^–3^) at each of the ambient monitoring locations (AMB1–AMB4; Table 3).

## 4 Environmental Implications

The field evidence collected during our study suggests an influence from the WtE plant at the nearest downwind ambient monitoring location (AMB3), 450 m downwind of the plant, substantiated by (i) an average downwind concentration increase of 0.04 ng m^–3^ for ∑PFAS_42_ and 0.017 ng m^–3^ for ∑EFSA_4_ and (ii) a distinct ambient air fingerprint of long-chain perfluorocarboxylic acids (PFCAs) at AMB3, closely resembling the emission fingerprints independently characterized at the 2 grate incinerator lines. Although the emission impact is measurable, concentration levels fell well below the current ambient air assessment framework of Flanders. Moreover, optimized AC dosage resulted in a quantifiable emission reduction of 90-95% at line 1. Open questions remain on the PFAS content (concentration and composition) of the treated municipal solid waste (input material) and the resulting destruction efficiency of the WtE plant. Waste streams are particularly heterogeneous and difficult to characterize for their PFAS content. Nevertheless, previous studies by Gehrmann et al.^56^ and Van Caneghem et al.^14^ reported high (>99.99%) destruction efficiencies for specific parent PFAS under municipal and hazardous waste incineration. Environmental PFAS management is currently challenged by the dual need for (i) standardized monitoring methods and harmonized regulation focused on dedicated target species of concern, while (ii) extensive screening studies are needed at different industries, to improve our understanding on the atmospheric fate of PFAS and identify relevant atmospheric target species for regulatory management policies.

The waste treatment sector represents a critical frontier for environmental management, as it may inadvertently act as a conduit for PFAS accumulation and potential environmental releases. Building experimental evidence will be key to closing this knowledge gap and ensuring comprehensive strategies for responsible PFAS management in the years to come.

## Supplementary Material



## Notes

Emission train schematics and target analytes (SI1), ambient target analytes (SI2), precipitation data (SI3), wind data (SI4), emission results for every individual campaign (SI5), QA/QC evaluation (SI6), applied mass flow rates and chimney characteristics (SI7), model results for the individual scenarios (SI8), and temporal concentration variability at the ambient monitoring locations (SI9) (PDF)
