# Supplementary material for: The Fate of Per- and Polyfluoroalkyl Substances (PFAS) in Municipal Solid Waste Incineration: Atmospheric Emissions from a Waste-to-Energy Plant
Source: Environ Sci Technol. 2026 Oct 20;60(43):30767–79. doi: 10.1021/acs.est.6c05879 (PMC13738988; doi:10.1021/acs.est.6c05879)

SUPPORTING INFORMATION

The Fate of Per- and Polyfluoroalkyl Substances (PFAS) in Municipal Solid Waste Incineration: Atmospheric Emissions from a Waste-to-Energy Plant

Jelle Hofman[1], Jan Peters[1], Griet Jacobs[2], Aline Reis De Carvalho[2], Gill Van den Bergh[2], Bart Baeyens[1], Wim Aerts[1], Jo Van Laer[1], Nic Moonen[1], Wouter Lefebvre[3], Patrick Berghmans[1], Stefan Voorspoels[2], Gert Otten[1]*

[1]*Air Quality & Industrial Emissions (AQIE) Team, Flemish Institute for Technological Research (VITO), Boeretang 200, 2400 Mol, Belgium*

[2]*Specialized Organic and Inorganic Analysis Lab (GOAL), Flemish Institute for Technological Research (VITO), Boeretang 200, 2400 Mol, Belgium*

[3]*Geology & Air Quality Modelling (GAM) Team, Flemish Institute for Technological Research (VITO), Boeretang 200, 2400 Mol, Belgium*

* Address: Vlasmeer 5, 2400 Mol, Belgium, Phone: +32 14 33 57 58, Email: jelle.hofman@vito.be, ORCID: 0000-0002-3450-6531

Pages: S1-S8

Tables: SI1, SI2, SI5, SI7, SI8

Figures: SI3, SI4, SI9

QA/QC information: SI6

SI1. Emission sampling train schematic (LUC/VI/003) and quantified PFAS compounds in the emission sampling method with experimentally derived recovery (%) and measurement uncertainties (U, k=2) (Hofman et al. 2025b) and experimentally derived LOQ levels in the analytical XAD2, filter and water fractions (LUC, 2025). A PFAS compound can be considered quantitative if recovery is within 70-120% and U<50%. Otherwise, the compound is considered indicative.

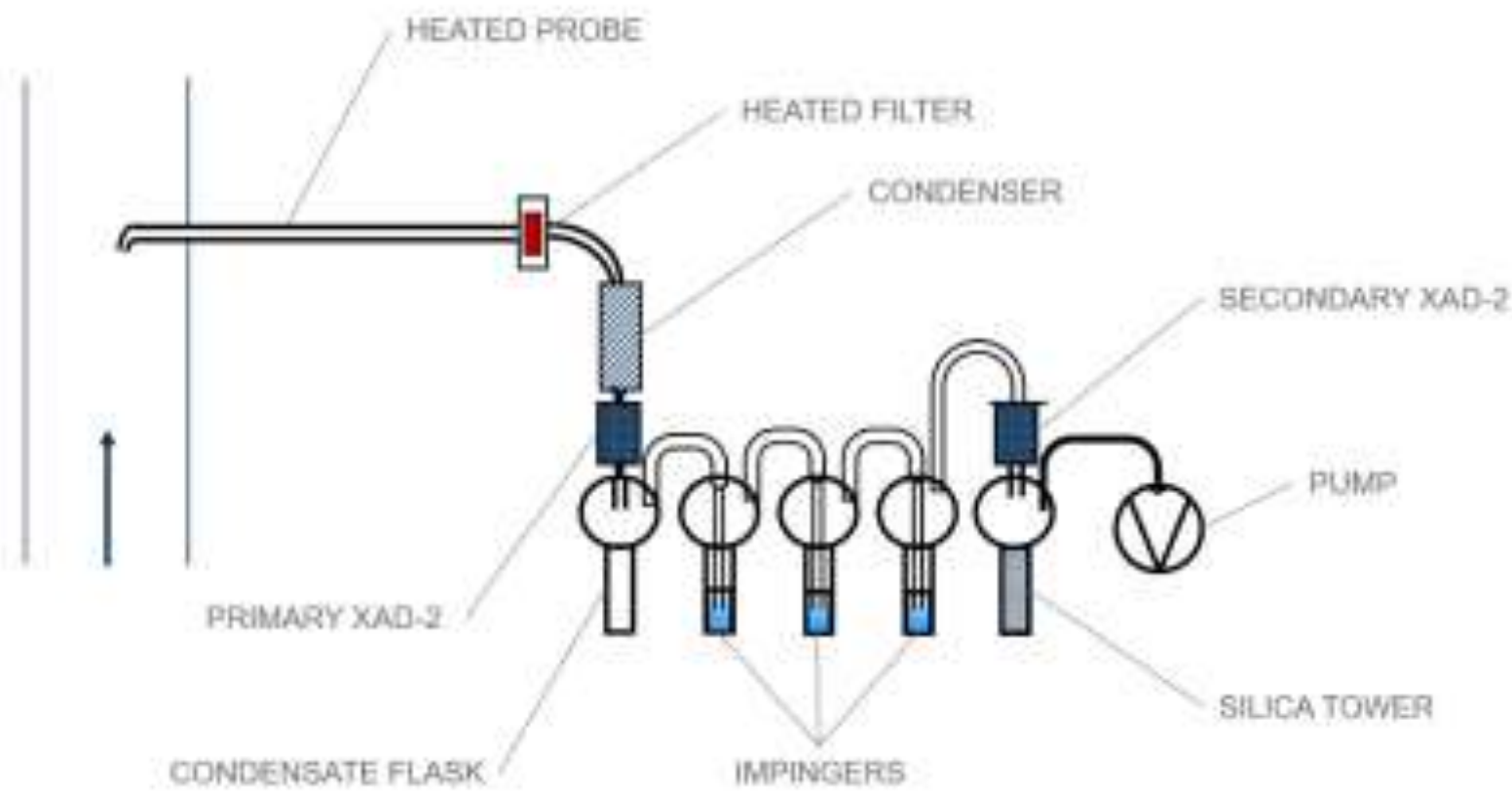

					LOQ		
					XAD2	Filter	Water
COMPOUND	CAS	RECOVERY (%)	U (k=2)	QUANT/IND	ng	ng	ng
PFBA	375-22-4	120%	34%	QUANT	ND	0.9	0.01
PFPeA	2706-90-3	100%	2%	QUANT	3	0.8	0.01
PFHxA	307-24-4	98%	10%	QUANT	1	1.3	0.01
PFHpA	375-85-9	93%	14%	QUANT	0.9	0.9	0.01
L-PFOA	335-67-1	91%	19%	QUANT	5	0.1	0.01
PFNA	375-95-1	92%	20%	QUANT	0.8	0.01	0.01
PFDA	335-76-2	99%	15%	QUANT	2	0.05	0.01
PFUnDA	2058-94-8	104%	28%	QUANT	0.2	0.04	0.01

**PFDoDA**	*307-55-1*	107%	33%	QUANT	0.5	0.04	0.01
**PFTrDA**	*72629-94-8*	109%	34%	IND	0.1	0.01	
**PFTeDA**	*376-06-7*	96%	50%	QUANT	0.3	0.01	0.01
**PFHxDA**	*67905-19-5*	80%	59%	IND	0.5	0.03	0.01
**PFODA**	*16517-11-6*	51%	117%	IND	ND	0.7	
**PFBS**	*375-73-5*	96%	7%	QUANT	0.4	0.8	
**PFPeS**	*2706-91-4*	91%	13%	QUANT	0.03	0.03	0.01
**L-PFHxS**	*355-46-4*	92%	15%	QUANT	0.05	0.05	0.01
**PFHpS**	*375-92-8*	86%	26%	QUANT	0.07	0.04	0.01
**L-PFOS**	*1763-23-1*	101%	8%	QUANT	3	0.8	0.01
**PFNS**	*68259-12-1*	90%	21%	QUANT	0.08	0.04	0.01
**PFDS**	*335-77-3*	85%	28%	QUANT	0.1	1.2	0.01
**PFUnDS**	*749786-16-1*	81%	34%	IND	0.09	0.03	
**PFDoDS**	*79780-39-5*	79%	36%	IND	0.03	0.7	
**PFTrDS**	*791563-89-8*	72%	51%	IND	0.02	0.02	
**4:2FTS**	*757124-72-4*	98%	3%	QUANT	0.02	0.4	0.01
**6:2FTS**	*27619-97-2*	93%	11%	QUANT	2.7	0.8	0.01
**8:2FTS**	*39108-34-4*	93%	22%	QUANT	0.1	0.5	0.01
**10:2FTS**	*120226-60-0*	108%	29%	IND	0.1	0.7	0.05
**PFBSA**	*30334-69-1*	100%	5%	QUANT	6	1.8	0.01
**MePFBSA**	*68298-12-4*	55%	140%	IND	ND	1.4	0.01
**MePFBSAA**	*159381-10-9*	103%	5%	QUANT	0.8	0.1	0.01
**PFHxSA**	*41997-13-1*	97%	14%	QUANT	0.4	0.8	0.01
**L-PFOSA**	*754-91-6*	56%	85%	IND	0.08	0.8	0.01
**L-MePFOSA**	*31506-32-8*	1%	273%	IND	0.2	1.4	0.01
**L-EtPFOSA**	*4151-50-2*	0%	289%	IND	ND	0.7	0.01
**L-MePFOSAA**	*2355-31-9*	94%	21%	QUANT	0.05	0.01	0.01
**L-EtPFOSAA**	*2991-50-6*	97%	22%	QUANT	0.05	0.7	0.01
**6:2diPAP**	*57677-95-9*	92%	47%	QUANT	0.09	0.005	
**6:2/8:2diPAP**	*943913-15-3*	81%	63%	IND	0.1	0.05	0.05
**8:2diPAP**	*678-41-1*	67%	72%	IND	0.7	0.08	0.01
**HFPO-DA**	*13252-13-6*	102%	8%	QUANT	3.5		0.01
**DONA**	*919005-14-4*	89%	17%	QUANT	0.1	0.02	0.01
**PFECHS**	*646-83-3*	88%	22%	QUANT	0.1	0.04	

SI2. Quantified PFAS compounds in the ambient air sampling method with associated measurement uncertainty (k=2; >50% = indicative) on filter and PUF samples

		**AMBIENT AIR (FILTER)**	**AMBIENT AIR (PUF)**

COMPOUND	ABBREVIATION	U (k=2)	REMARK	U (k=2)	REMARK
perfluor-n-butaanzuur<br>perfluoro-n-butanoic acid	PFBA	<50%		<50%	
perfluor-n-pentaanzuur<br>perfluoro-n-pentanoic acid	PFPeA	<50%		<50%	
perfluor-n-hexaanzuur<br>perfluoro-n-hexanoic acid	PFHxA	<50%		<50%	
perfluor-n-heptaanzuur<br>perfluoro-n-heptanoic acid	PFHpA	<50%		<50%	
perfluor-n-octaanzuur (lineair)<br>perfluoro-n-octanoic acid (linear)	L-PFOA	<50%		<50%	
perfluoroctaanzuur (totaal)<br>perfluorooctanoic acid (total)	T-PFOA	-		-	
perfluor-n-nonaanzuur<br>perfluoro-n-nonanoic acid	PFNA	<50%		<50%	
perfluor-n-decaanzuur<br>perfluoro-n-decanoic acid	PFDA	<50%		<50%	
perfluor-n-undecaanzuur<br>perfluoro-n-undecanoic acid	PFUnDA	<50%		<50%	
perfluor-n-dodecaanzuur<br>perfluoro-n-dodecanoic acid	PFDoDA	<50%		<50%	
perfluor-n-tridecaanzuur<br>perfluoro-n-tridecanoic acid	PFTrDA	<50%		<50%	indicatief/<br>indicative
perfluor-n-tetradecaanzuur<br>perfluoro-n-tetradecanoic acid	PFTeDA	<50%		<50%	
perfluor-n-hexadecaanzuur<br>perfluoro-n-hexadecanoic acid	PFHxDA	<50%		<50%	indicatief/<br>indicative
perfluor-n-octadecaanzuur<br>perfluoro-n-octadecanoic acid	PFODA	>50%	indicatief/<br>indicative	>50%	indicatief/<br>indicative
perfluor-n-butaansulfonzuur<br>perfluoro-n-butanesulfonic acid	PFBS	<50%		<50%	
perfluor-n-pentaansulfonzuur<br>perfluoro-n-pentanesulfonic acid	PFPeS	<50%		<50%	
perfluor-n-hexaansulfonzuur (lineair)<br>perfluoro-n-hexanesulfonic acid (linear)	L-PFHxS	<50%		<50%	
perfluorhexaansulfonzuur (totaal)<br>perfluorohexanesulfonic acid (total)	T-PFHxS	-		-	
perfluor-n-heptaansulfonzuur<br>perfluoro-n-heptanesulfonic acid	PFHpS	<50%		<50%	
perfluor-n-octaansulfonzuur (lineair)<br>perfluoro-n-octanesulfonic acid (linear)	L-PFOS	<50%		<50%	
perfluoroctaansulfonzuur (totaal)<br>perfluorooctanesulfonic acid (total)	T-PFOS	-		-	
perfluor-n-nonaansulfonzuur<br>perfluoro-n-nonanesulfonic acid	PFNS	<50%		<50%	indicatief/<br>indicative
perfluor-n-decaansulfonzuur<br>perfluoro-n-decanesulfonic acid	PFDS	<50%		<50%	indicatief/<br>indicative
perfluor-n-undecaansulfonzuur<br>perfluoro-n-undecanesulfonic acid	PFUnDS	<50%		<50%	indicatief/<br>indicative
perfluor-n-dodecaansulfonzuur<br>perfluoro-n-dodecanesulfonic acid	PFDoDS	<50%		<50%	indicatief/<br>indicative
perfluor-n-tridecaansulfonzuur<br>perfluoro-n-tridecanesulfonic acid	PFTrDS	<50%		<50%	indicatief/<br>indicative
4:2 fluortelomeersulfonzuur<br>4:2 fluorotelomer sulfonic acid	4:2 FTS	>50%	indicatief/<br>indicative	>50%	
8:2 fluortelomeersulfonzuur<br>8:2 fluorotelomer sulfonic acid	8:2 FTS	>50%	indicatief/<br>indicative	>50%	indicatief/<br>indicative

10:2 fluortelomeersulfonzuur<br>10:2 fluorotelomer sulfonic acid	10:2 FTS	>50%	indicatief/<br>indicative	>50%	indicatief/<br>indicative
perfluor-n-butaansulfonamide<br>perfluoro-n-butanesulfonamide	PFBSA	<50%		<50%	
N-methylperfluor-n-butaansulfonamide<br>N-methylperfluoro-n-butanesulfonamide	MePFBSA	<50%		<50%	indicatief/<br>indicative
2-N-methylperfluor-n-butaansulfonamide azijnzuur<br>2-(N-methylperfluoro-n-butanesulfonamido)acetic acid	MePFBSAA	<50%		<50%	indicatief/<br>indicative
perfluor-n-hexaansulfonamide (lineair)<br>perfluoro-n-hexanesulfonamide (linear)	L-PFHxSA	<50%		<50%	
perfluor-n-hexaansulfonamide (totaal)<br>perfluoro-n-hexanesulfonamide (total)	T-PFHxSA	-		-	
perfluor-n-octaansulfonamide (lineair)<br>perfluoro-n-octanesulfonamide (linear)	L-PFOSA	<50%		<50%	
perfluoroctaansulfonamide (totaal)<br>perfluorooctanesulfonamide (total)	T-PFOSA	-		-	
N-methylperfluor-n-octaansulfonamide (lineair)<br>N-methylperfluor-n-ooctanesulfonamide (linear)	L-MePFOSA	<50%		<50%	indicatief/<br>indicative
N-methylperfluoroctaansulfonamide (totaal)<br>N-methylperfluorooctanesulfonamide (total)	T-MePFOSA	-		-	indicatief/<br>indicative
N-ethylperfluor-n-octaansulfonamide (lineair)<br>N-ethylperfluoro-n-octanesulfonamide (linear)	L-EtPFOSA	<50%			indicatief/<br>indicative
N-ethylperfluoroctaansulfonamide (total)<br>N-ethylperfluorooctanesulfonamide (total)	T-EtPFOSA	-		-	indicatief/<br>indicative
2-(N-methylperfluor-n-octaansulfonamido)azijnzuur (lineair)<br>2-(N-methylperfluoro-n-octanesulfonamido)acetic acid (linear)	L-MePFOSAA	<50%		<50%	
2-(N-methylperfluoroctaansulfonamido)azijnzuur (totaal)<br>2-(N-methylperfluorooctanesulfonamido)acetic acid (total)	T-MePFOSAA	-		-	
2-(N-ethylperfluor-n-octaansulfonamido)azijnzuur (lineair)<br>2-(N-ethylperfluoro-n-octanesulfonamido)acetic acid (linear)	L-EtPFOSAA	<50%		<50%	
2-(N-ethylperfluor-n-octaansulfonamido)azijnzuur (totaal)<br>2-(N-ethylperfluoro-n-octanesulfonamido)acetic acid (total)	T-EtPFOSAA	-		-	
6:2 fluortelomeerfosfaat diester<br>6:2 fluorotelomerphosphate diester	6:2 diPAP	<50%		<50%	indicatief/<br>indicative
6:2/8:2 fluortelomeerfosfaat diester<br>6:2/8:2 fluorotelomerphosphate diester	6:2/8:2 diPAP	<50%		<50%	indicatief/<br>indicative
8:2 fluortelomeerfosfaat diester<br>8:2 fluorotelomerphosphate diester	8:2 diPAP	<50%		<50%	indicatief/<br>indicative
perfluor-2-propoxypropaanzuur<br>perfluoro-2-propoxypropanoic acid	HFPO-DA	<50%		<50%	
4,8-dioxa-3H-perfluornonaanzuur<br>4,8-dioxa-3H-perfluorononanoic acid	ADONA	<50%		<50%	
perfluor-4-ethylcyclohexaansulfonzuur<br>perfluoro-4-ethylcyclohexane sulfonic acid	PFECHS	<50%		<50%	

SI3. Measured precipitation (mm) at the Stabroek station during the ambient monitoring campaign (7/9/2023 - 30/11/2023)

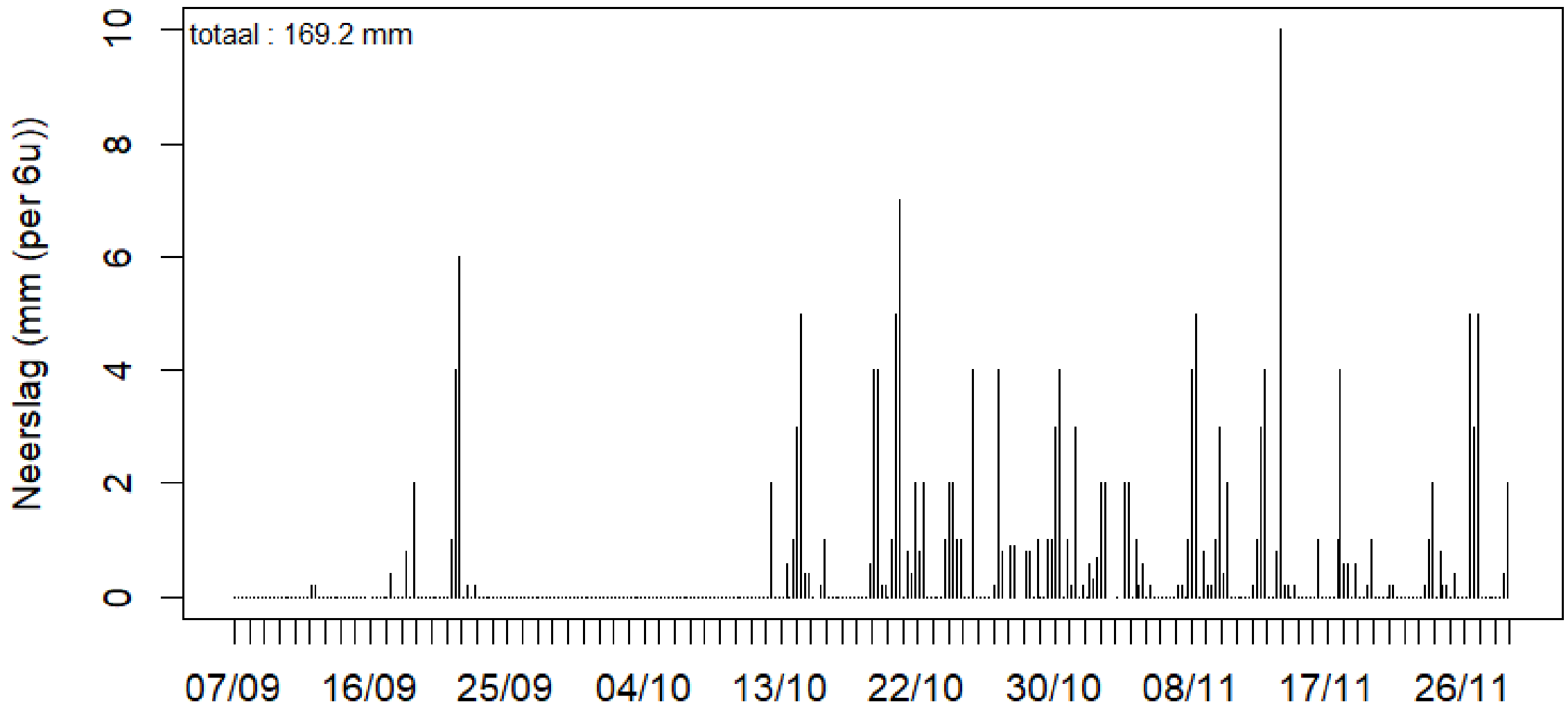

SI4. Observed wind distributions (source: RMI station Deurne) during each of the consecutive monitoring periods between 7/9/2023 and 30/11/2023 (P1-P6 in Table 1).

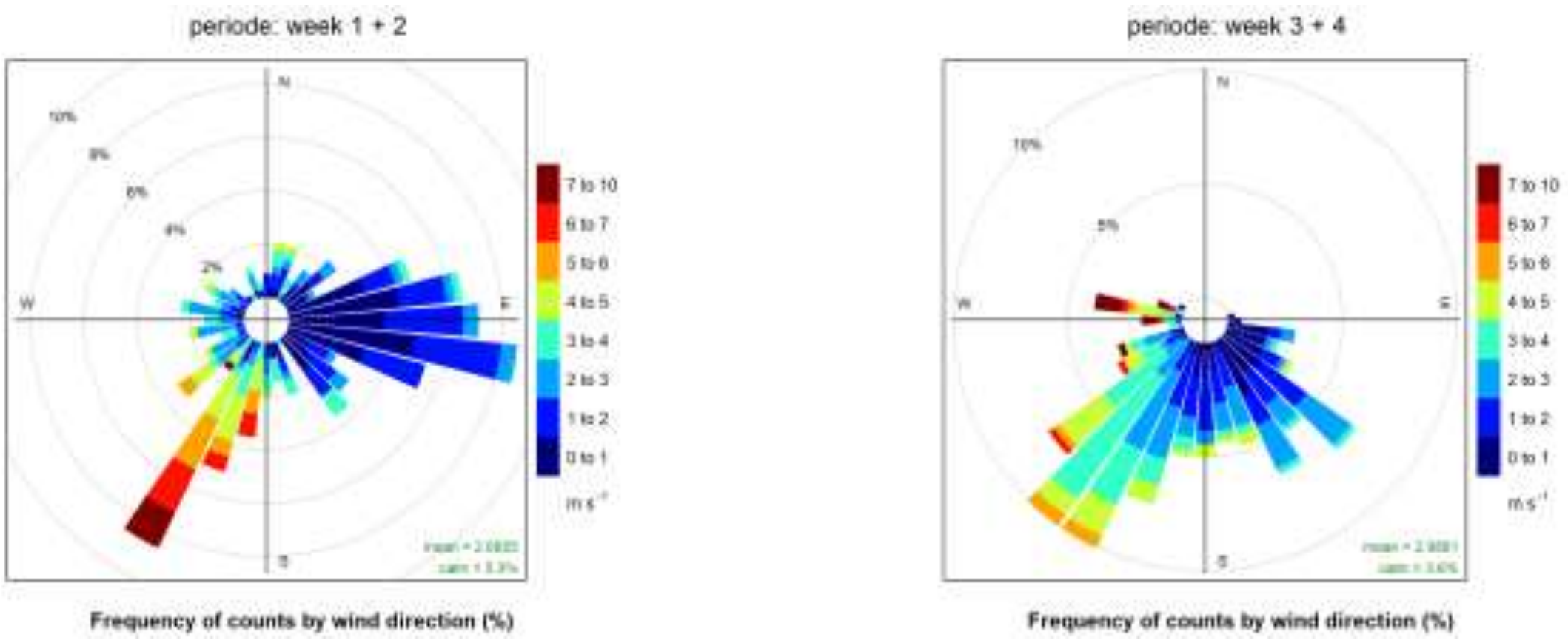

periode: week 5 + 6

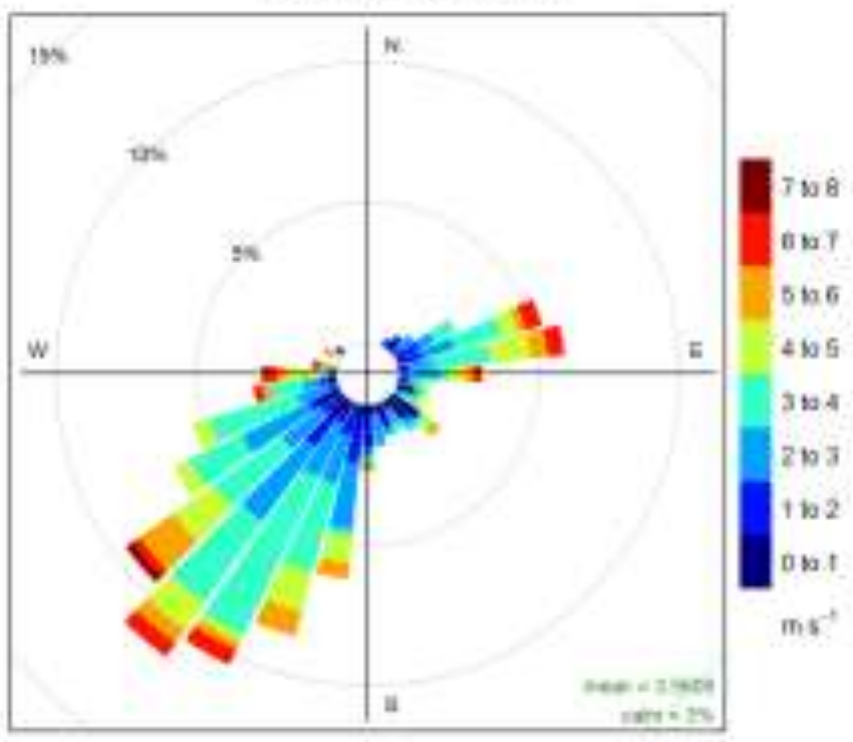

Frequency of counts by wind direction (%)

periode: week 7 + 8

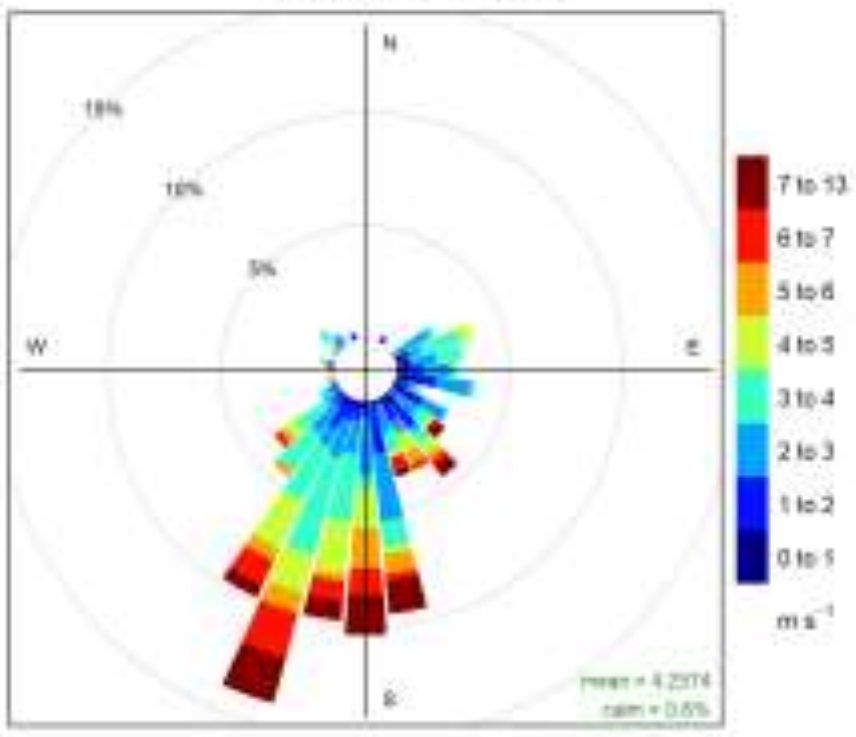

Frequency of counts by wind direction (%)

periode: week 9 + 10

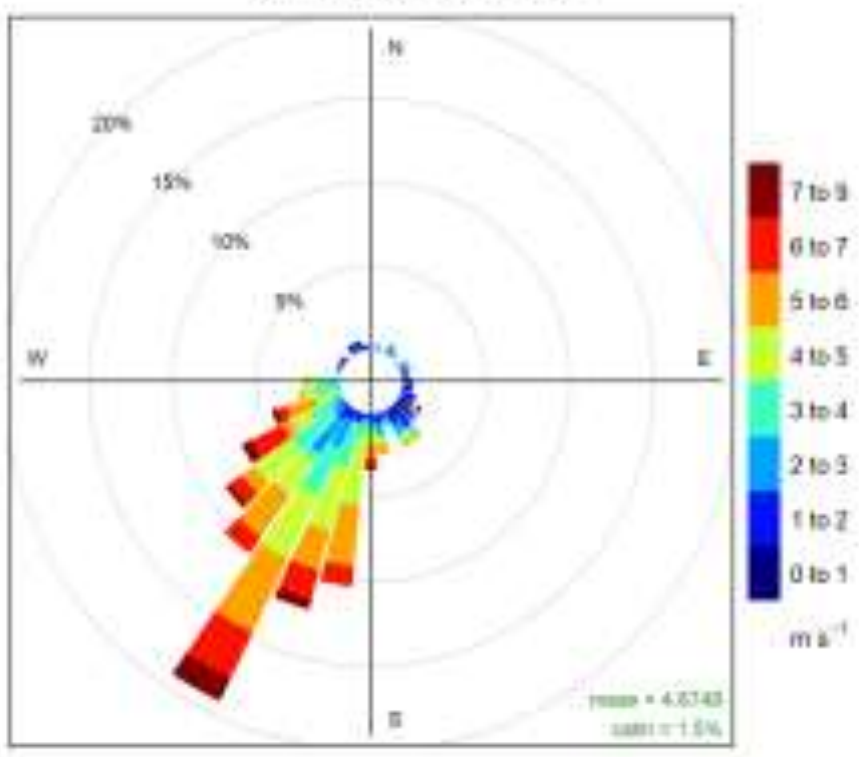

Frequency of counts by wind direction (%)

periode: week 11 + 12

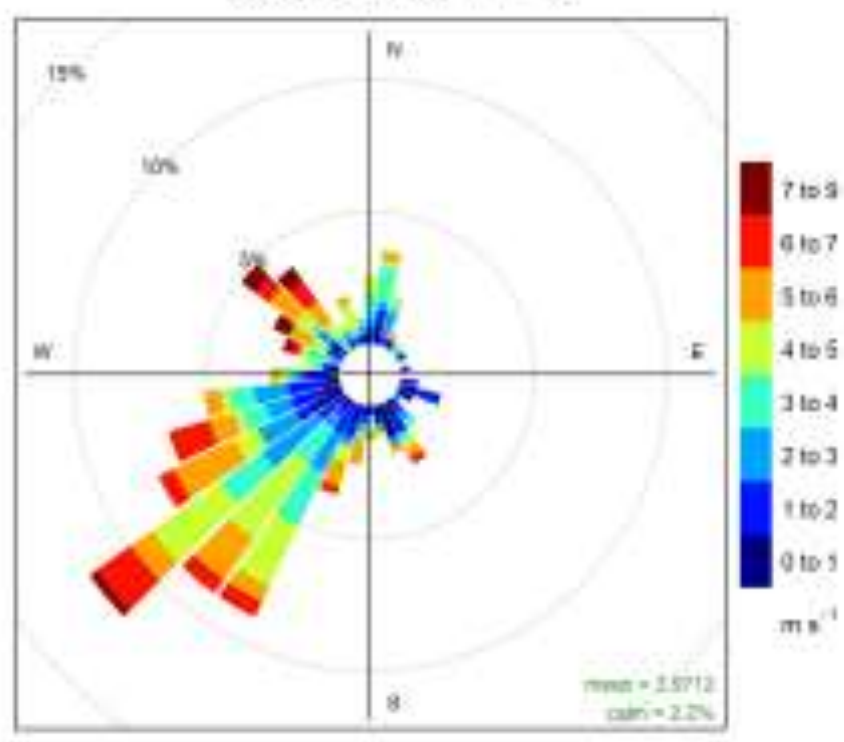

Frequency of counts by wind direction (%)

SI5. Normalized emission concentrations (ng $Nm^{-3}dr$ at 11% reference O_2) for each individual compound and $\sum PFAS_{42}$ and $\sum EFSA_4$ sum (<LOQ=0). Compounds marked with an asterisk (*) have increased measurement uncertainty and should be considered indicative. The orange-shaded compounds (T-) represent linear (L-) and branched isomers combined. Red values approximate field blank concentrations (measured value <10x field blank concentration)

	14/04/2023		04/05/2023		23/08/2023		12/10/2023
	Line 1	Line 2	Line 1	Line 2	Line 1	Line 2	Line 1
COMPOUND	ng/Nm³dr	ng/Nm³dr	ng/Nm³dr	ng/Nm³dr	ng/Nm³dr	ng/Nm³dr	ng/Nm³dr
PFBA	53.2	6.0	34.0	5.7	15.2	17.8	16.8
PFPeA	44.1	1.9	53.0	1.1	2.7	3.6	3.1
PFHxA	55.0	0.9	67.4	1.3	3.2	5.3	1.6
PFHpA	73.9	2.9	95.1	1.0	2.8	7.7	1.7
L-PFOA	102.6	5.8	119.5	3.7	0.5	1.4	3.6
T-PFOA	101.4	5.6	119.5	3.7	0.5	1.5	3.6
PFNA	253.5	9.3	163.0	3.4	2.4	16.4	7.1
PFDA	314.2	11.1	187.2	4.2	1.6	23.2	7.6
PFUnDA	413.8	15.8	238.7	9.8	8.7	28.5	9.3
PFDoDA	502.4	16.4	254.9	14.1	9.0	33.7	20.1
PFTrDA*	693.6	34.9	256.2	25.6	25.9	44.2	31.2
PFTeDA	750.7	23.7	245.7	41.6	45.7	41.8	20.6
PFHxDA*	668.6	37.6	177.2	<LOQ	74.3	23.2	7.1
PFODA*	111.8	4.4	240.5	<LOQ	6.9	2.4	1.2
PFBS	1.0	0.4	0.9	0.8	0.5	0.3	0.1
PFPeS	<LOQ	<LOQ	<LOQ	<LOQ	0.6	0.5	<LOQ
L-PFHxS	0.2	0.3	0.2	0.2	2.8	2.0	0.1
T-PFHxS	0.3	0.3	0.3	0.4	3.5	2.6	0.1
PFHpS	<LOQ	<LOQ	<LOQ	<LOQ	0.4	0.3	<LOQ
L-PFOS	0.7	0.7	9.4	11.5	0.5	0.7	0.3
T-PFOS	1.4	1.5	14.1	16.9	0.7	1.1	0.4
PFNS	<LOQ	<LOQ	<LOQ	<LOQ	<LOQ	<LOQ	<LOQ
PFDS	<LOQ	<LOQ	<LOQ	<LOQ	<LOQ	<LOQ	<LOQ
PFUnDS*	<LOQ	<LOQ	<LOQ	<LOQ	<LOQ	<LOQ	<LOQ
PFDoDS*	<LOQ	<LOQ	<LOQ	<LOQ	<LOQ	<LOQ	<LOQ

**PFTrDS**	<LOQ	<LOQ	<LOQ	<LOQ	<LOQ	<LOQ	<LOQ
**4:2FTS**	<LOQ	<LOQ	<LOQ	<LOQ	<LOQ	<LOQ	<LOQ
**6:2FTS**	0.2	0.2	0.2	0.2	0.4	0.3	1.4
**8:2FTS**	0.1	<LOQ	<LOQ	<LOQ	<LOQ	<LOQ	<LOQ
**10:2FTS***	<LOQ	<LOQ	<LOQ	<LOQ	<LOQ	<LOQ	<LOQ
**PFBSA**	0.1	0.1	<LOQ	<LOQ	<LOQ	<LOQ	<LOQ
**MePFBSA***	<LOQ	<LOQ	<LOQ	<LOQ	<LOQ	<LOQ	<LOQ
**MePFBSAA**	<LOQ	<LOQ	0.1	0.1	<LOQ	0.1	0.2
**PFHxSA**	<LOQ	<LOQ	<LOQ	<LOQ	<LOQ	<LOQ	<LOQ
**L-PFOSA***	<LOQ	<LOQ	<LOQ	<LOQ	<LOQ	<LOQ	<LOQ
**T-PFOSA***	<LOQ	<LOQ	<LOQ	<LOQ	<LOQ	<LOQ	<LOQ
**L-MePFOSA***	<LOQ	<LOQ	<LOQ	<LOQ	<LOQ	<LOQ	<LOQ
**T-MePFOSA***	<LOQ	<LOQ	<LOQ	<LOQ	<LOQ	<LOQ	<LOQ
**L-EtPFOSA***	<LOQ	<LOQ	<LOQ	<LOQ	<LOQ	<LOQ	<LOQ
**T-EtPFOSA***	<LOQ	<LOQ	<LOQ	<LOQ	<LOQ	<LOQ	<LOQ
**L-MePFOSAA**	<LOQ	<LOQ			<LOQ	<LOQ	<LOQ
**T-MePFOSAA**	<LOQ	<LOQ	<LOQ	<LOQ	<LOQ	<LOQ	<LOQ
**L-EtPFOSAA**	<LOQ	<LOQ	<LOQ	<LOQ	<LOQ	<LOQ	<LOQ
**T-EtPFOSAA**	<LOQ	<LOQ	<LOQ	<LOQ	<LOQ	<LOQ	<LOQ
**6:2diPAP***	<LOQ	<LOQ			<LOQ	<LOQ	<LOQ
**6:2/8:2diPAP***	<LOQ	<LOQ	<LOQ	<LOQ	<LOQ	<LOQ	<LOQ
**8:2diPAP***	<LOQ	<LOQ	<LOQ	<LOQ	<LOQ	<LOQ	<LOQ
**HFPO-DA**	9.8	3.3	<LOQ	<LOQ	0.3	0.5	<LOQ
**ADONA**	<LOQ	<LOQ	18.5	4.8	0.1	0.1	0.1
**PFECHS**	<LOQ	<LOQ	0.3	0.3	<LOQ	<LOQ	<LOQ
**$\sum PFAS_{42}$**	**4049.2**	**176.4**	**2167.1**	**135.1**	**205.5**	**255.0**	**133.2**
**$\sum EFSA_{4}$**	**356.7**	**16.7**	**296.9**	**24.4**	**7.1**	**21.5**	**11.2**

SI6. Evaluation of LUC/VI/003 method quality assurance and quality controls (QA/QC's) of the conducted emission measurements in SI5.

Sampling standard (SS) recoveries for PFBA, PFOA and PFOS (criterion = 50-130%) were rather low for the first campaign in April (35-66%) and satisfactory for all other campaigns in May (62-147%), August (87-103%) and October (77-86%).

Sampling train breakthrough at the second XAD-2 module (criterion = <30%) was never observed.

Medium blank levels were generally low, except for the August campaign, where limits of quantification (LOQ) were raised (2x field blank concentration) for some sulfonic acids (C3 to C8 PFSA; PFPrS to PFOS) due to presence in the medium XAD2 blanks. However, the impact of this contamination is considered negligible since the reported emissions mainly consist of carboxyl acids.

The **field blank criterion** (<10% of field sample concentration) was breached for several compounds (red values in SI5), indicating that emission concentrations approximate field blank levels.

SI7. Calculated mass flow rates (upper table) and considered chimney characteristics (lower table) for $\sum PFAS_{42}$ and $\sum EFSA_4$ in the emission scenario modelling.

**MASS FLOW RATE (mg h^{-1})**					
**SCENARIO 1: AVERAGE before optimization line 1**			**SCENARIO 1: MAX before optimization line 1**		
**$\sum PFAS_{42}$**	140	mg h^{-1}	**$\sum PFAS_{42}$**	173	mg h^{-1}
**$\sum EFSA_4$**	15	mg h^{-1}	**$\sum EFSA_4$**	15	mg h^{-1}
**SCENARIO 2: AVERAGE all measurements**			**SCENARIO 2: MAX all measurements**		
**$\sum PFAS_{42}$**	78	mg h^{-1}	**$\sum PFAS_{42}$**	173	mg h^{-1}
**$\sum EFSA_4$**	8	mg h^{-1}	**$\sum EFSA_4$**	15	mg h^{-1}
**SCENARIO 3: AVERAGE after optimization line 1**			**SCENARIO 3: MAX after optimization line 1**		
**$\sum PFAS_{42}$**	15	mg h^{-1}	**$\sum PFAS_{42}$**	18	mg h^{-1}
**$\sum EFSA_4$**	1	mg h^{-1}	**$\sum EFSA_4$**	2	mg h^{-1}

	**LINE 1**	**LINE 2**
**Lambert coordinates X (m)**	150579	150581
**Lambert coordinates Y (m)**	204179	204181
**Height (m)**	60	60
**Diameter (m)**	1.4	1.4
**Temperature (°C)**	63,8	65,3
**Average Flow rate (Nm³ h^{-1})**	43212	40811

SI8. Modelled yearly average and maximum daily-average $\sum PFAS_{42}$ and $\sum EFSA_4$ concentrations (ng m^{-3}) for each of the considered emission scenarios (1-3).

SCENARIO 1: AVERAGE before optimization line 1			SCENARIO 1: MAX before optimization line 1		
$\sum PFAS_{42}$	0.025	ng m^{-3}	$\sum PFAS_{42}$	0.192	ng m^{-3}
$\sum EFSA_{4}$	0.003	ng m^{-3}	$\sum EFSA_{4}$	0.017	ng m^{-3}
**SCENARIO 2: AVERAGE all measurements**			**SCENARIO 2: MAX all measurements**		
$\sum PFAS_{42}$	0.014	ng m^{-3}	$\sum PFAS_{42}$	0.192	ng m^{-3}
$\sum EFSA_{4}$	0.0014	ng m^{-3}	$\sum EFSA_{4}$	0.017	ng m^{-3}
**SCENARIO 3: AVERAGE after optimization line 1**			**SCENARIO 3: MAX after optimization line 1**		
$\sum PFAS_{42}$	0.0027	ng m^{-3}	$\sum PFAS_{42}$	0.02	ng m^{-3}
$\sum EFSA_{4}$	0.0002	ng m^{-3}	$\sum EFSA_{4}$	0.002	ng m^{-3}

SI9. Temporal variability of ambient $\sum PFAS_{42}$ and $\sum EFSA_4$ concentration (ng m^{-3}) at the considered ambient monitoring locations (AMB1-AMB4).

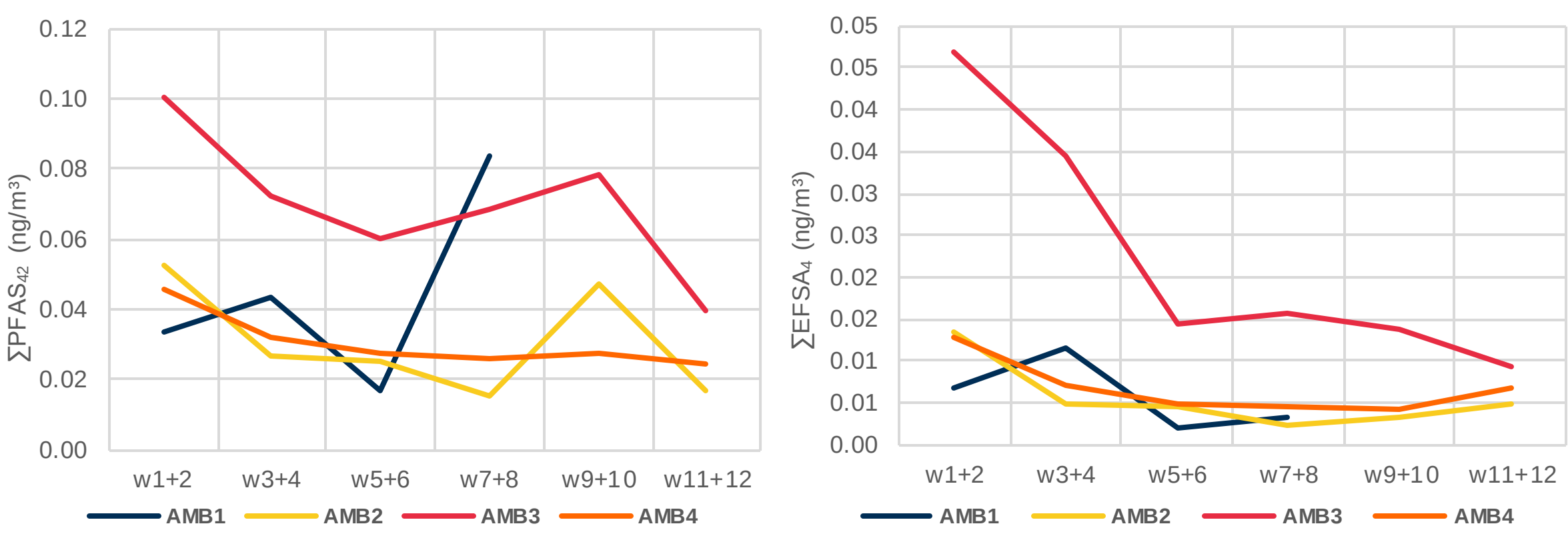

Supplement: Supplementary file 1 [file es6c05879_si_001.pdf]
